# Evolutionary Inferences Based on Thoracic Limb Muscles of South American Coati (*Nasua nasua*): Brachium and Craniolateral Antebrachium

**DOI:** 10.1111/ahe.70182

**Published:** 2026-10-16

**Authors:** Juan Fernando Vélez García, Roseâmely Angélica de Carvalho Barros, Maria Angelica Miglino

**Affiliations:** ^1^ Research Group of Medicine and Surgery in Small Animals, Department of Animal Health, Faculty of Veterinary Medicine and Animal Science Universidad del Tolima Ibagué Colombia; ^2^ Postgraduate Program in Anatomy of the Domestic and Wild Animals, Department of Surgery, School of Veterinary Medicine and Animal Science Universidade de São Paulo São Paulo Brazil; ^3^ Laboratory of Comparative Anatomy of Wild Animals, Department of Biological Sciences, Institute of Biotechnology Universidade Federal de Catalão Catalão Brazil; ^4^ University of Marilia—UNIMAR Marília Brazil

**Keywords:** anatomical variations, artery, forelimb, myology, nerve

## Abstract

The South American coati ( *Nasua nasua* ) is a mammal of the order Carnivora and family Procyonidae, characterized by scansorial locomotion. Accordingly, several anatomical and functional studies examined its thoracic limbs. Nevertheless, the anatomical descriptions and terminology differ among those studies, and none considered the evolutionary derivations of the brachium and antebrachium muscles based on topology, innervation and anatomical variants. Therefore, the present study aimed to infer the muscle derivations and reconstruct muscle maps of the brachial and craniolateral antebrachial muscles in *N. nasua* based on the evidence from direct gross dissections and comparisons with literature in embryology and myology of representative mammals from monotremes to procyonids. The caudomedial group will be addressed in a separate manuscript. Seventeen thoracic limbs from eight formaldehyde‐fixed specimens and one fresh specimen of *N. nasua* were dissected. As main results that differed from the available literature, the cranial part of the tensor fasciae antebrachii muscle of non‐*Nasua* procyonids was not lost in *N. nasua* but fused to the caudal part. The *anconeus medialis* muscle was not a head of the *triceps brachii* muscle and may have derived from the caudomedial antebrachial musculature. The *caput accessorium* of the *triceps brachii* muscle may have derived mainly from its *caput laterale*. The *extensor carpi radialis* muscle was undivided proximally and only separated into two bellies (*longus* and *brevis*) from the middle third of the antebrachium. In conclusion, the brachium and craniolateral antebrachium muscles of *N. nasua* had several differences from those described previously, and most evolutionary derivations could be inferred.

## 1 Introduction

The South American coati ( *Nasua nasua* ) is a mammal belonging to the order Carnivora, suborder Caniformia and family Procyonidae (Hassanin et al. 2021; Nyakatura and Bininda‐Emonds 2012). The genus *Nasua* (coatis) diverged approximately 17 million years ago from a common ancestor with the extant genera *Procyon* (raccoons), *Bassariscus* (ringtails) and *Bassaricyon* (olingos) within the family Procyonidae (Hassanin et al. 2021; Nyakatura and Bininda‐Emonds 2012). *Nasua nasua* is geographically restricted to South America (Emmons and Helgen 2016) and exhibits mainly gregarious and diurnal habits (Hirsch 2022). This species is considered scansorial due to its combined terrestrial and arboreal abilities (Gardin et al. 2021; McClearn 1992). It frequently uses its thoracic limbs to dig for invertebrates in the soil and to shred dead logs (Alves‐Costa et al. 2004; Beisiegel 2001; Hirsch 2022; McClearn 1992). It also climbs trees to feed on fruits (Alves‐Costa et al. 2004; Beisiegel 2001; Hirsch 2022) and occasionally to construct nests (Olifiers et al. 2009) or escape from predators (Beisiegel 2001). Owing to this distinctive locomotion, gross anatomical studies of the thoracic limb muscles have been conducted in this species compared with other carnivorans (Böhmer et al. 2020; Mackintosh 1875; McClearn 1985; Meckel 1829; Santos, Bertassoli, Oliveira, et al. 2010; Tarquini et al. 2023). In this way, Tarquini et al. (2023) observed a high development of the elbow flexor muscles in *N. nasua* , particularly due to a high mass of a craniolateral antebrachial muscle, such as the *m. brachioradialis*, which was associated with its more specialized climbing abilities than other species. The increased flexibility of the elbow has been potentially associated with an adaptation for locomotion in variable habits (Dangerfield et al. 2026). To act on the elbow, the *triceps brachii*, *anconeus*, *tensor fasciae antebrachii* and *anconeus medialis* muscles locate caudally in the brachium and extend the elbow, while the biceps brachii and brachialis muscles locate cranially in the brachium and act as elbow flexors (Hermanson et al. 2020; Vélez‐García, de Carvalho Barros, and Miglino 2023). Besides, some craniolateral antebrachial muscles support the elbow flexion, such as the *brachioradialis* and *extensor carpi radialis* muscles (Hermanson et al. 2020). Nevertheless, several differences are evident among the anatomical descriptions of these muscles in *N. nasua* (Böhmer et al. 2020; Mackintosh 1875; McClearn 1985; Meckel 1829; Santos, Bertassoli, Oliveira, et al. 2010; Tarquini et al. 2023), particularly in the terminology employed, the reported attachments and the identification of anatomical variants. These include the use of former names for the muscles that act on the elbow (e.g., *m. epitrochlearis* for *m. tensor fasciae antebrachii*), variations in the insertions of biceps brachii and brachialis muscles, and differences in the reported number of heads of *m. triceps brachii* (among three and five heads). Previously, the embryonic studies reported that the dorsal and ventral division of the myotome gave rise to the elbow flexors and extensors, respectively (Diogo et al. 2018). However, the more recent embryological study observed that the *m. brachialis* (an elbow flexor muscle) derives from the antebrachium subdivision of the dorsal division together with the craniolateral antebrachial muscles (Smith‐Paredes et al. 2022). This last group acts mainly on the carpal extension, digital extension and supination, and its anatomical descriptions also differ among authors in *N. nasua* (Böhmer et al. 2020; Mackintosh 1875; McClearn 1985; Meckel 1829; Santos, Bertassoli, Oliveira, et al. 2010; Tarquini et al. 2023).

Evolutionary studies to establish muscle homology among chordates use topology, innervation and embryology to infer the derivation of the muscles (Diogo et al. 2018). However, the most available studies in *N. nasua* only considered the topology to describe the brachium and craniolateral antebrachium muscles (Böhmer et al. 2020; Mackintosh 1875; McClearn 1985; Meckel 1829; Santos, Bertassoli, Oliveira, et al. 2010; Tarquini et al. 2023). Whilst recent studies in other procyonids found important intra‐ and interspecific differences in the evolutionary knowledge of the thoracic limb muscles based on the topology, innervation, arterial distribution, anatomical variants and available literature (Perdomo‐Cárdenas et al. 2021; Vélez García et al. 2024, 2025; Vélez‐García, Carrión Blanco, et al. 2023; Vélez‐García and Miglino 2023). Among the interspecific differences in procyonids, the *m. tensor fasciae antebrachii* divides into two parts (pars cranialis and pars caudalis) in different non‐*Nasua* procyonids (Vélez García et al. 2024). The *caput accessorium* of *m. triceps brachii* is proposed to be derived mainly from the *caput laterale* due to the distribution of the radial nerve (Vélez García et al. 2024). The *m. anconeus medialis* (formerly named *m. anconeus epitrochlearis* or *m. epitrochleoanconeus*) is independent of the *m. triceps brachii* and could evolutionarily have derived from the *m. flexor carpi ulnaris* since it is innervated by the ulnar nerve (Vélez García et al. 2024; Vélez‐García, Carrión Blanco, et al. 2023). One study performed directly in the shoulder muscles of *N. nasua* corroborated that the *m. articularis humeri* is not homologous to the *m. coracobrachialis* nor *m. coracobrachialis brevis* because these have different innervation and topology in the shoulder joint (Vélez García et al. 2025). Thus, the present study aimed to describe the anatomy of brachium and craniolateral antebrachium muscles in *N. nasua* to infer the evolutionary derivations based on the topology, innervation, anatomical variants and available literature. Furthermore, the arterial distribution to these muscles will be described to observe possible associations with the muscle separation during muscular development. The caudomedial antebrachium muscles will be addressed in a separate manuscript.

## 2 Materials and Methods

### 2.1 Bioethical and Environmental Licences

This research was approved by the bioethics committees of the Universidade de São Paulo (CEUAx agreement number 3928240820) and Universidade Federal de Catalão (CEUA‐UFCAT agreement number 01/22). The cadavers had been collected with the environmental licence number 37072‐2 (System of Authorization and Information in Biodiversity—SISBIO) from roads in Goiás State (Brazil). The wildlife care centre of CORPOCALDAS (Corporación Autónoma Regional de Caldas, environmental authority of Caldas Department, Colombia) donated a dead *N. nasua* specimen to the Universidad del Tolima (Colombia) for anatomical research.

### 2.2 Specimens and Location of the Research

Fifteen thoracic limbs from eight formaldehyde‐fixed carcasses of *N. nasua* that had been used in previous investigations of their brachial plexus (Felipe 2014; Vélez‐García, de Carvalho Barros, and Miglino 2023) and shoulder muscles (Vélez García et al. 2025; Vélez‐García and Miglino 2023) were used. Both thoracic limbs from one specimen without formalin fixation were also used, having a total sample of seventeen limbs. Each specimen was identified in order of dissection (NnS1–NnS9), and their right and left thoracic limbs were identified as RTL and LTL, respectively (NnS1‐RTL/LTL–NnS9‐RTL/LTL) based on Vélez García et al. (2025) (Table 1). The first specimen (NnS1) was dissected at the Laboratorio de Anatomía Veterinaria of Universidad del Tolima (UT‐LAV, Tolima, Colombia), the second to eighth (NnS2–NnS8) at the Laboratório de Anatomia Comparativa de Animais Silvestres of Universidade Federal de Catalão (UFCAT‐LACRAS), and the last one (NnS9) at the Laboratório de Biologia Integrativa e Conservação of Universidade Federal de Catalão (UFCAT‐LBIC, Goiás, Brazil). The humerus, ulna and radius of an adult male of *N. nasua* (UFCAT‐MAM 218) from the latter laboratory were used to reconstruct the muscle maps.

**TABLE 1:** Identification of specimens and their dissected limbs.

Species	ID	Laboratory	Origin	Cause of death	Sex	Age	Limb	ID Limb
*Nasua nasua*	NnS1	UT‐LAV	Caldas (Colombia)	Unknown	M	I	RTL	NnS1‐RTL
							LTL	NnS1‐LTL
	NnS2	UFCAT‐LACRAS	Goiás (Brazil)	Roadkill	F	A	RTL	NnS2‐RTL
							LTL	NnS2‐LTL
	NnS3	UFCAT‐LACRAS	Goiás (Brazil)	Roadkill	M	sA	RTL	NnS3‐RTL
							LTL	NnS3‐LTL
	NnS4	UFCAT‐LACRAS	Goiás (Brazil)	Roadkill	M	A	RTL	NnS4‐RTL
							LTL	NnS4‐LTL
	NnS5	UFCAT‐LACRAS	Goiás (Brazil)	Roadkill	M	J	RTL	NnS5‐RTL
							LTL	NnS5‐LTL
	NnS6	UFCAT‐LACRAS	Goiás (Brazil)	Roadkill	F	J	RTL	NnS6‐RTL
							LTL	NnS6‐LTL
	NnS7	UFCAT‐LACRAS	Goiás (Brazil)	Roadkill	U	J	RTL	NnS7‐RTL
							LTL	NnS7‐LTL
	NnS8	UFCAT‐LACRAS	Goiás (Brazil)	Roadkill	F	J	LTL	NnS8‐LTL
	NnS9	UFCAT‐LBIC	Goiás (Brazil)	Roadkill	M	sA	RTL	NnS9‐RTL
							LTL	NnS9‐LTL

Abbreviations: A, adult; F, female; I, infant; ID Limb, identification of the limb; ID, specimen identification; J, juvenile; LTL, left thoracic limb; M, male; RTL, right thoracic limb; sA, sub‐adult; U, unknown sex; UFCAT‐LACRAS, Universidade Federal de Catalão—Laboratório de Anatomia Comparativa de Animais Silvestres; UFCAT‐LBIC, Universidade Federal de Catalão—Laboratório de Biologia Integrativa e Conservação; UT‐LAV, Universidad del Tolima—Laboratorio de Anatomía Veterinaria.

### 2.3 Dissection

The muscle architecture classification (fusiform, unipennate, bipennate, multipennate and flat) was based on gross observation and fleshy fibres direction in longitudinal sections of the muscle belly in all cases. Muscle attachments (origin and insertion) were categorized as fleshy (direct observation of muscle bands attached to the structure), tendinous (via a tendon) or aponeurotic (via a flat tendon). The attachment sites of muscles, as well as the shoulder and elbow joint capsules and elbow ligaments, were mapped onto the bones. Carcasses previously used in brachial plexus studies (Felipe 2014; Vélez‐García, de Carvalho Barros, and Miglino 2023) were re‐examined to obtain a more detailed description of the nerve branches distribution to the muscles, as myological studies in mammals have demonstrated the usefulness of innervation patterns for muscle identification. This re‐examination did not compromise the muscle observations. Additionally, because three specimens (NnS1, NnS2 and NnS3) received successful repletion arterial in their thoracic limbs after injection with natural latex tinctured with red vinyl via femoral, the arterial supply for the muscles was described. Photographs of gross dissections of the first specimen (NnS1) were taken with a Canon EOS 6D camera equipped with a Canon 100 mm macro lens, and the other dissections with a Canon T5i camera equipped with a Canon 60 mm macro lens.

### 2.4 Anatomical Terminology and Comparation With Other Studies

The anatomical terms for the descriptions were mainly based on the International Committee on Veterinary Gross Anatomical Nomenclature at the *Nomina Anatomica Veterinaria* (NAV) (International Committee on Veterinary Gross Anatomical Nomenclature 2017). On the other hand, we used other terms that were suggested in evolutionary studies in procyonids, such as *m. anconeus lateralis* (AL) and *m. anconeus medialis* (AM) (Vélez García et al. 2024; Vélez‐García, Carrión Blanco, et al. 2023). The results were compared with previous studies in the genus *Nasua* (Böhmer et al. 2020; Mackintosh 1875; McClearn 1985; Meckel 1829; Santos, Bertassoli, Oliveira, et al. 2010; Tarquini et al. 2023). The writing of the manuscript was reviewed using two artificial intelligence‐based tools (Copilot and Rubriq).

## 3 Results

### 3.1 Cranial Muscles of Brachium

#### 3.1.1 *m. biceps brachii* (BB)

The BB was a bipennate muscle that originated via a tendon from the supraglenoid tubercle, which passed internally to the shoulder joint capsule and through the intertubercular groove of the humerus where it was protected by the transverse humeral retinaculum (THR) (Figure 1). It inserted via a flat tendon onto the caudolateral surface of the radial tuberosity. There was a synovial bursa (*bursa bicipitoradialis*) between its tendon and radial tuberosity (Figures 1 and 2). The proximal muscular branch of the musculocutaneous nerve (McN) innervated it. The transverse cubital (TCA) and superficial brachial (SBA) arteries supplied it distally (Figure 3, Table 2), while the proximal and middle supply was variant among specimens. In two cases bilaterally (NnS1 and NnS2), a branch of the subscapular artery (SbA) and two branches of the brachial artery (bicipital arteries) supplied it. There were anatomical variants in its arterial supply: In one limb (NnS2‐RTL), one branch of the cranial circumflex humeral artery (CrCHA) supplied it. In two limbs (NnS4 bilaterally), four bicipital arteries supplied it (Figure 3), but the more proximal bicipital artery was homologous to that formed branch from the SbA of other specimens (NnS1 and NnS2).

**FIGURE 1:**
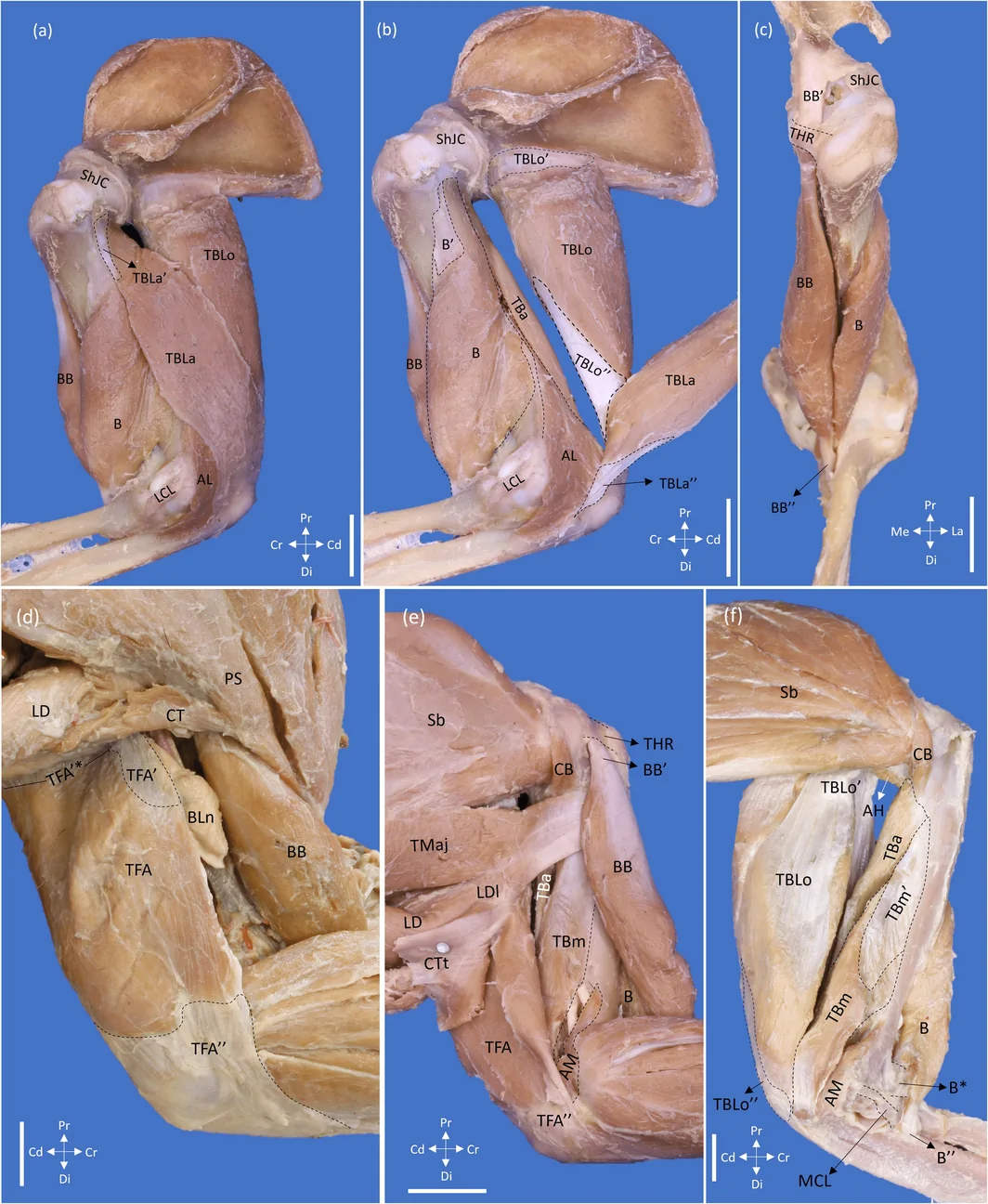
Brachium muscles of *Nasua nasua* (left thoracic limbs). (a) Superficial lateral view; (b) superficial lateral view where the caput laterale of *m. triceps brachii* was displaced caudally; (c) cranial view; (d) medial superficial view; (e) tensor fasciae antebrachii and medial belly of *m. latissimus dorsi* were displaced caudally; (f) biceps brachii, tensor fasciae antebrachii, latissimus dorsi and teres major muscles were removed. AH, *m. articularis humeri*; AL, *m. anconeus lateralis*; AM, *m. anconeus medialis*; B*, fascia joining the belly of B to the elbow joint capsule; B, *m. brachialis*; B′, aponeurotic origin of B; B″, insertion tendon of B; BB, *m. biceps brachii*; BB’, origin tendon of BB; BB″, insertion tendon of BB; BLn, brachial lymph node; CB, *m. coracobrachialis*; Cd, caudal; Cr, cranial; CT, *m. cutaneus trunci*; CTt, common insertion tendon of medial belly of *latissimus dorsi*, *pectoralis abdominalis*, *cutaneus trunci* and *pectoralis profundus* muscles; Di, distal; La, lateral; LCL, lateral collateral ligament of elbow; LD, *m. latissimus dorsi*; LDl, lateral belly of LD; MCL, medial collateral ligament of elbow; Me, medial; Pr, proximal; PS, *mm. pectorales superficiales*; Sb, *m. subscapularis*; ShJC, shoulder joint capsule; TB, *m. triceps brachii*; TBa, *caput accessorium* of TB; TBLa, *caput laterale* of TB; TBLa’, aponeurotic origin of TBLa; TBLa”, aponeurotic insertion of TBLa; TBLo, *caput longum* of TB; TBLo’, origin tendon of TBLo; TBLo”, insertion tendon of TBLo; TBm, *caput mediale* of TB; TBm’, tendinous origin of TBm; TFA, *m. tensor fasciae antebrachii*; TFA’*, fleshy origin of TFA; TFA’, aponeurotic origin of TFA; TFA”, aponeurosis of insertion of TFA; THR, transverse humeral retinaculum; TMaj, *m. teres major*. Scale bars: 10 mm.

**FIGURE 2:**
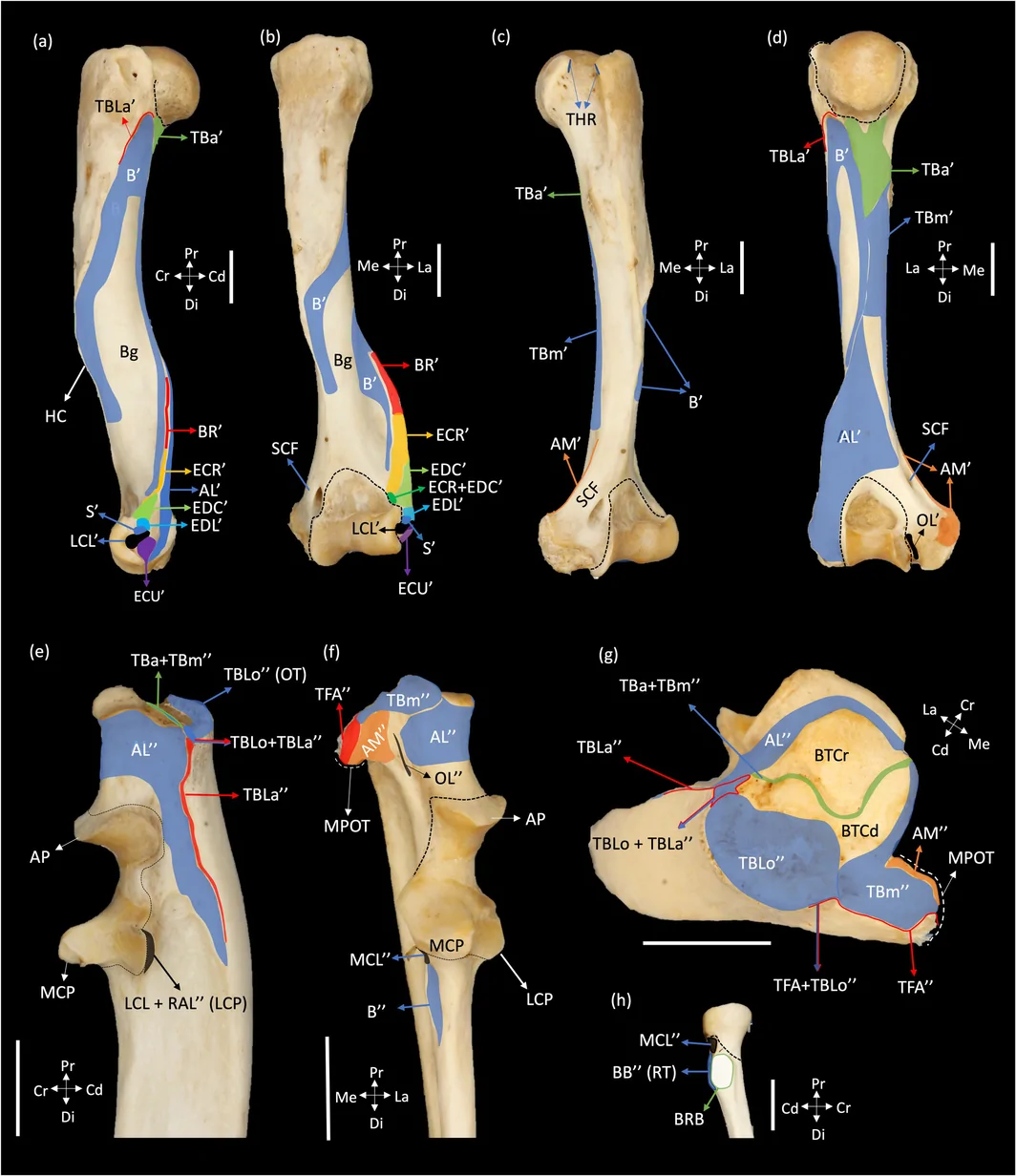
Muscle mapping of *Nasua nasua* , left humerus (a–d), ulna (e–g) and radius (h). Lateral (a), cranial (b), craniomedial (c) and caudal (d) views of humerus. Lateral (e), craniomedial (f) and proximal (g) views of ulna. Medial view of radius (h). AL, *m. anconeus lateralis*; AM, *m. anconeus medialis*; AP, anconeus process; B, *m. brachialis*; BB, *m. biceps brachii*; Bg, groove of B (*sulcus m. brachialis*); BR, *m. brachioradialis*; BRB, bicipitoradial bursa (*bursa bicipitoradialis*); BTCd, *bursa tricipitalis caudalis* (caudal tricipital bursa;) Cd, caudal; BTCr, *bursa tricipitalis cranialis* (cranial tricipital bursa); Cr, cranial; Di, distal; ECR + EDC, origin common tendon of ECR and EDC; ECR, *m. extensor carpi radialis*; ECU, *m. extensor carpi ulnaris*; EDC, *m. extensor digitorum communis*; EDL, *m. extensor digitorum lateralis*; HC, humeral crest; La, lateral; LCL + RAL, common insertion of LCL and RAL; LCL, lateral collateral ligament of elbow; LCP, lateral coronoid process of ulna; MCL, medial collateral ligament of elbow; MCP, medial coronoid process of ulna; Me, medial; MPOT, medial process of olecranon tuberosity; OL, olecranon ligament; OT, olecranon tuberosity; Pr, proximal; RAL, radial annular ligament; RT, radial tuberosity; S, *m. supinator*; SCF, supracondylar foramen; TB, *m. triceps brachii*; TBa + TBm, common insertion of TBa and TBm; TBa, *caput accessorium* of TB; TBLa, *caput laterale* of TB; TBLa+TBLo, common insertion of TBLa and TBLo; TBLo, *caput longum* of TB; TBm, *caput mediale* of TB; TFA + TBLo, common insertion of TFA and TBLo; TFA, *m. tensor fasciae antebrachii*; THR, transverse humeral retinaculum; TI, trochlear incisure. Dashed lines: limits of the shoulder and elbow joint capsules. ‘, origin; ”, insertion. Scale bars: 10 mm.

**FIGURE 3:**
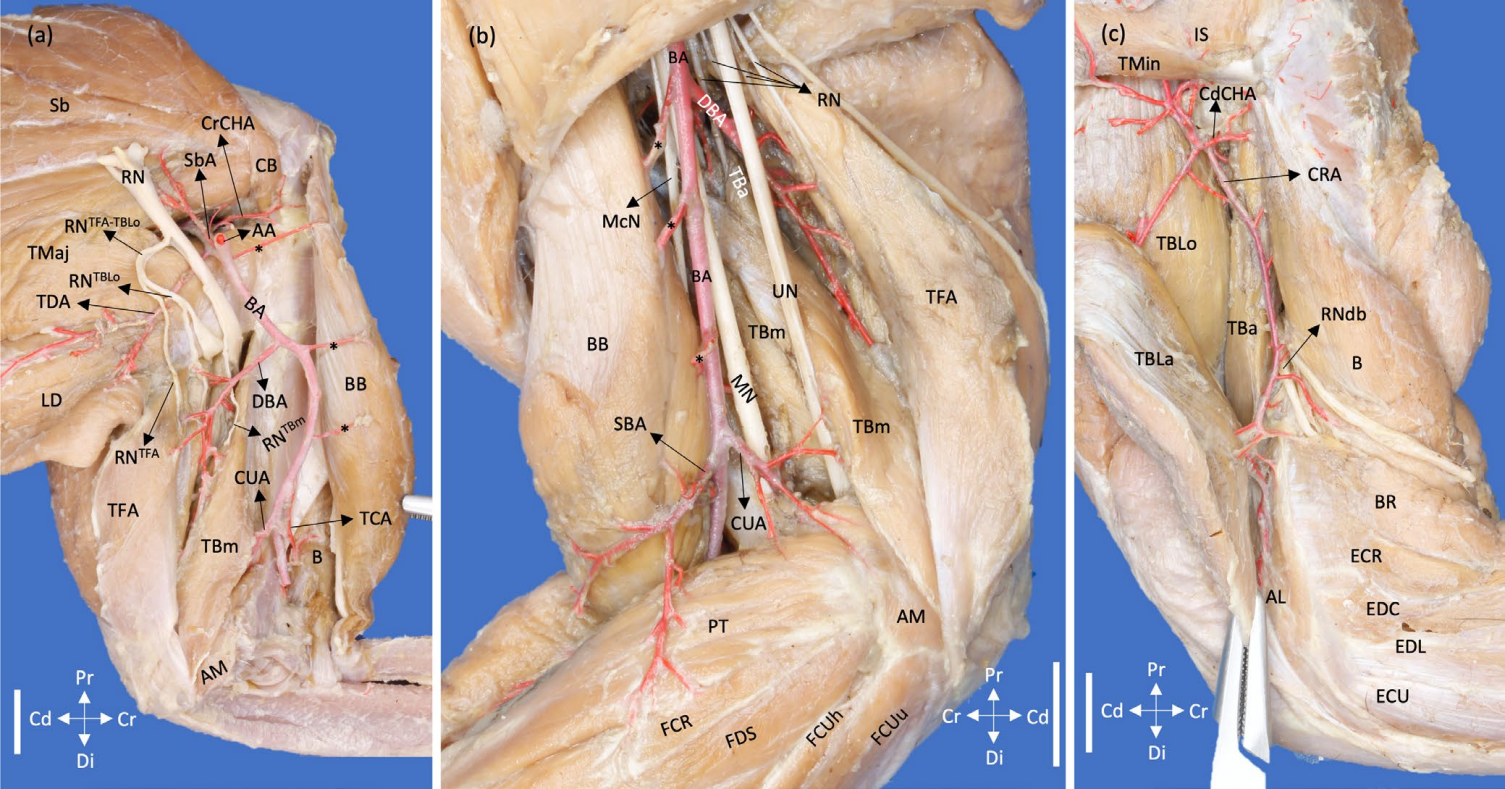
Arterial supply and innervation to the intrinsic muscles of shoulder and brachium muscles in *Nasua nasua* . (a) Medial view of a left thoracic limb; (b) medial and (c) lateral views of a right thoracic limb. AA, axillary artery (*a. axillaris*); AL, *m. anconeus lateralis*; AM, *m. anconeus medialis*; B, *m. brachialis*; BA, brachial artery (*a. brachialis*); BB, *m. biceps brachii*; BR, *m. brachioradialis*; CB, *m. coracobrachialis*; CdCHA, caudal circumflex humeral artery (*a. circumflexa humeri caudalis*); CRA, collateral radial artery (*a. collateralis radialis*); CrCHA, cranial circumflex humeral artery (*a. circumflexa humeri cranialis*); CUA, collateral ulnar artery (*a. collateralis ulnaris*); DBA, deep brachial artery (*a. profunda brachii*); DSA, dorsal scapular artery (*a. scapularis dorsalis*); ECR, *m. extensor carpi radialis*; ECU, *m. extensor carpi ulnaris*; EDC, *m. extensor digitorum communis*; EDL, *m. extensor digitorum lateralis*; FCR, *m. flexor carpi radialis*; FCUh, *m. flexor carpi ulnaris caput humerale*; FCUu, *m. flexor carpi ulnaris caput ulnare*; FDS, *m. flexor digitorum superficialis*; IS, *m. infraspinatus*; LD, *m. latissimus dorsi*; McN, musculocutaneous nerve (*n. musculocutaneus*); MN, median nerve (*n. medianus*); MN, median nerve (*N. medianus*); PT, *m. pronator teres*; RN, Radial nerve (*n. radialis*); RNdp, deep branch of RN (*n. radialis ramus profundus*); RN^TBLO^, branch of RN to TBLo; RN^TBm^, branch of RN to TBm; RN^TFA^, branch of RN to TFA; RN^TFA‐TBLo^, common branch of RN to TFA and TBLo; Sb, *m. subscapularis*; Sb, *m. subscapularis*; SbA, subscapular artery (*a. subscapularis*); SBA, superficial brachial artery (*a. brachialis superficialis*); TB, *m. triceps brachii*; TBa, *caput accessorium* of TB; TBLa, *caput laterale* of TB; TBLo, *caput longum* of TB; TBm, *caput mediale* of TB; TCA, transverse cubital artery (*a. transversa cubiti*); TDA, thoracodorsal artery (*a. thoracodorsalis*); TFA, *m. tensor fasciae antebrachii*; TMaj, *m. teres major*; TMin, *m. teres minor*; UN, ulnar nerve (*n. ulnaris*). *Bicipital arteries (*aa. bicipitales*). Scale bars: 10 mm.

**TABLE 2:** Summary of characteristics of brachium muscles in *Nasua nasua* , and main differences between the present study and other studies in the genus *Nasua*.

Muscle (abbreviation)	Shape	Origin	Insertion	Innervation and arterial supply	Differences with other studies
*m. biceps brachii* (BB)	Bipennate	Supraglenoid tubercle of scapula	Radial tuberosity	Proximal muscular branch of musculocutaneous nerve, transverse cubital, superficial brachial and bicipital (between two and four branches) arteries.	Additional insertion onto the ulnar tuberosity (Santos, Bertassoli, Oliveira, et al. 2010).
					Additional small tendon to *m. brachialis* (Meckel 1829)
*m. brachialis* (B)	Bipennate	Lateral and caudal surfaces of humeral diaphysis without origin from the sulcus of *m. brachialis* (Inverted ‘V’ shape in muscles maps)	Medial surface of ulna, distal to medial collateral ligament of elbow	Distal muscular branch of musculocutaneous nerve, collateral radial and transverse cubital arteries.	Additional origin from the sulcus of *m. brachialis* (No inverted ‘V’ shape in the muscles maps) (Tarquini et al. 2023)
		Almost along lateral supracondylar crest			Proximal surface of lateral supracondylar crest (Tarquini et al. 2023)
					Additional insertion onto the radial tuberosity (Santos, Bertassoli, Oliveira, et al. 2010)
			Strong fascia joining the B belly to cranial aspect of elbow joint capsule		No reported
*m. tensor fasciae antebrachii* (TFA)	Flat	*m. latissimus dorsi pars lateralis* and the common tendon of several extrinsic thoracic limb muscles.	Tendon of *m. triceps brachii caput longum* (TBLo), caudomedial margin of olecranon and antebrachial fascia	Radial nerve, deep brachial and brachial arteries.	Origin from *m. latissimus dorsi* and *m. cutaneus trunci* (Tarquini et al. 2023). Unique origin from the *m. latissimus dorsi* (Mackintosh 1875). No insertion onto TBLo tendon or fusion with this (Böhmer et al. 2020; Mackintosh 1875; Santos, Bertassoli, Oliveira, et al. 2010; Tarquini et al. 2023).
		Variant: Additional origin from TMaj (six cases)			Additional origin from TMaj in *N. narica* (Mackintosh 1875)
		Variant: Shared fibres with *m. latissimus dorsi pars lateralis* (one case)			Shared fibres with LD in all cases (Tarquini et al. 2023).
			Fusion with insertion tendon of TBLo		No tendinous fusion of its insertion tendon with TBLo (Böhmer et al. 2020; Mackintosh 1875; Santos, Bertassoli, Oliveira, et al. 2010; Tarquini et al. 2023)
*m. triceps brachii* (TB)	Multipennate (four heads)	Scapula and humerus	Olecranon tuberosity	Radial nerve, caudal circumflex humeral, collateral radial, collateral ulnar and deep brachial arteries	Three heads (no caput accessorium) (Böhmer et al. 2020; Mackintosh 1875; Meckel 1829)
					Five heads (adding AM, Tarquini et al. 2023)
			Presence of two tricipital bursae in its tendons of insertion		No reported
*Caput longum* of *m. triceps brachii* (TBLo)	Bipennate	Ventral third of the caudal margin of scapula (including infraglenoid tubercle)	Caudal surface of olecranon tuberosity	Radial nerve, caudal circumflex humeral, collateral radial and deep brachial arteries	It is not very separated from TBLa (Mackintosh 1875).
					No fleshy origin inside the tendon of origin of TBLo.
					Two tendons of origin in *N. narica* (Mackintosh 1875).
*Caput laterale* of *m. triceps brachii* (TBLa)	Unipennate	Via an aponeurosis from the lateral aspect of humeral neck, tricipital line and brachial fascia over B	Olecranon tuberosity via a common with TBLo and independent onto caudolateral margin of the olecranon	Radial nerve, caudal circumflex humeral and collateral radial arteries	Origin via fleshy fibres and shared fibres with B and the tendon of origin of BB (Tarquini et al. 2023)
					Additional insertion onto olecranon via fleshy fibres together with TBLo (Tarquini et al. 2023)
		Variant: Partial fusion with TBa (one limb)			TBLa fused to TBm (taking into account that Mackintosh 1875 should consider TBa within TBm).
*Caput accessorium* of *m. triceps brachii* (TBa)	Bipennate	Humeral neck and the proximal fourth of caudal surface of humeral shaft	Via a common tendon with TBm onto the cranial aspect of the olecranon tuberosity	Radial nerve, collateral radial and deep brachial arteries	No origin from the humeral shaft (Santos, Bertassoli, Oliveira, et al. 2010; Tarquini et al. 2023)
					No described *caput* (Böhmer et al. 2020; Mackintosh 1875; Meckel 1829).
*Caput mediale* of *m. triceps brachii* (TBm)	Unipennate	Medial surface of humerus between the origin of TBa (at the level of insertion of TMaj and LD) and proximal to supracondylar foramen	Cranial and medial aspects of the olecranon tuberosity	Radial nerve, deep brachial and collateral ulnar arteries	In the muscle maps of humerus its origin is lesser and distal to the common insertion of the TMaj and LD muscles (Tarquini et al. 2023)
*m. anconeus lateralis* (AL)	Pyramidal	Distal half of humerus (lateral supracondylar crest and lateral epicondyle) and origin tendon of *m. extensor carpi ulnaris* (ECU)	Cranial and lateral surfaces of olecranon	Radial nerve, collateral ulnar, collateral radial and recurrent cranial interosseous arteries	No origin from lateral epicondyle and tendon of origin of ECU, and no insertion onto the cranial aspect of the olecranon (Tarquini et al. 2023)
					Additional origin from the medial epicondyle (Santos, Bertassoli, Oliveira, et al. 2010)
*m. anconeus medialis* (AM)	Triangular	Supracondylar foramen and medial epicondyle of humerus	Medial surface and distal margin of a process of olecranon tuberosity	Ulnar nerve and collateral ulnar artery	Reported with other names: ‘*caput accessorium*’ (Böhmer et al. 2020) or ‘*caput mediale accessorium* of *m. triceps brachii* (*m. epitrochleo‐anconeus*, *m. anconeus internus*)’ (Tarquini et al. 2023)
					Unique origin from the supracondylar foramen (Mackintosh 1875; Tarquini et al. 2023)
					No reported muscle (Meckel 1829; Santos, Bertassoli, Oliveira, et al. 2010).
					No additional insertion via tendinous fibres (Tarquini et al. 2023)

#### 3.1.2 *m. brachialis* (B)

The B was bipennate and originated from along the lateral and caudal surfaces of the humeral shaft, forming the shape of an inverted ‘V’ in the humeral muscle maps (Figure 2). The origin from the caudal surface extended from the lateral aspect of the humeral neck to almost along the medial aspect of the lateral supracondylar crest. The origin from the lateral surface extended from the humeral neck to the humeral crest. The origin was via tendon fibres in the caudal aspect to the deltoid tuberosity, tricipital line and humeral neck. The origin was fleshy in the medial aspect of the caudal surface of the humeral shaft, caudal aspect of the humeral crest (medial to the common tendon of insertion of the pectorales superficiales and cleidobrachialis muscles) and medial aspect of the lateral supracondylar crest (Figures 1 and 2). There was no origin from the middle surface of the groove of *m. brachialis* of the humerus (*sulcus m. brachialis*) due to its constant bipennate shape (as shown in Figure S1). A strong fascia adhered the belly of B to the cranial aspect of the elbow joint capsule (Figure 1). The B inserted via a tendon onto the distal surface to the medial coronoid process of the ulna, just distal to the insertion of the medial collateral ligament of the elbow (Figures 1 and 2). The distal muscular branch of the musculocutaneous nerve (McN), collateral radial artery (CRA), and TCA supplied it (Figure 3; Table 2). As an anatomical variant, in one specimen bilaterally (NnS1), it also inserted via fleshy fibres onto the adjacent proximal and medial aspects to its insertion tendon (Table 3).

**TABLE 3:** Summary of anatomical variations of the brachium and craniolateral antebrachium muscles of *Nasua nasua* .

Muscle (abbreviation)	Anatomical variations of muscles	Proportion
**Brachium**		
*m. brachialis* (B)	Insertion via fleshy fibres onto proximal‐ and medially adjacent to its tendinous insertion.	2/17
*m. tensor fasciae antebrachii* (TFA)	Origin from the *m. teres major* via tendinous fibres	6/17
	Origin from the *m. teres major* via fleshy fibres	3/17
	Shared fleshy fibres with *m. latissimus dorsi* in its origin	1/17
	Insertion onto *caput mediale* of *m. triceps brachii*	1/17
*m. triceps brachii* (TB)	*Caput laterale* partially fused with the *caput accessorium*	1/17
	Accessory *caput laterale* with origin from the lateral supracondylar crest.	1/17
*m. anconeus lateralis* (AL)	Additional insertion onto the tendon of *caput accessorium* of *m. triceps brachii*	1/17
	Additional insertion onto the common tendon of *caput laterale* and *caput longum of m. triceps brachii*	1/17
**Craniolateral antebrachium**		
*m. brachioradialis* (BR)	Shared fleshy fibres with the *m. extensor carpi radialis* proximally.	2/17
	Shared fleshy fibres with the *m. brachialis* proximally through which passed the deep branch of the radial nerve.	1/17
*m. extensor digitorum communis* (EDC)	Additional origin from the elbow joint capsule	1/17
	The belly only formed three tendons where the most medial tendon formed tendons to digits II and III	1/17
*m. abductor digiti I longus* (AbDIL)	Additional origin from the lateral collateral ligament of elbow	2/17
*m. extensor digiti I et II* (EDI‐II)	Origin distal to the lateral coronoid process of the ulna	1/17

### 3.2 Caudal Muscles of Brachium

#### 3.2.1 *m. tensor fasciae antebrachii* (TFA)

The TFA was a flat muscle of parallel fibres located on the caudal and medial surfaces of the tricipital region. It originated via fleshy fibres from the *m. latissimus dorsi* (LD) *pars lateralis* (LDl) and via aponeurotic fibres from the common tendon of *cutaneus trunci* (CT), *pectoralis profundus* (PP), *pectoralis abdominalis* and *latissimus dorsi pars medialis* (LDm) muscles. The muscle close to the elbow formed an aponeurosis that fused to the caudal aspect of the insertion tendon of *m. triceps brachii caput longum* (TBLo) and also inserted independently onto the caudomedial margin of olecranon (caudal to *m. triceps brachii caput mediale* [TBm]) and antebrachial fascia (Figure 1). The radial nerve (RN) innervated it via a common branch to TBLo (RN^TFA‐TBLo^) (Figure 3). The deep brachial artery (DBA) (Figure 3) and a small branch from the brachial artery (BA) supplied it (Table 2). A brachial lymph node was observed at the medial surface of the brachium between TFA and BB (Figure 1). As anatomical variants (Table 3), the TFA additionally originated from the *m. teres major* (TMaj) via tendinous fibres in six cases (NnS6‐RTL, NnS8‐LTL and bilaterally in NnS2 and NnS7) and via fleshy fibres in three cases (NnS6‐LTL and bilaterally in NnS1). In one case (NnS3‐LTL), the TFA shared a few fibres with LDl in its origin. In two cases bilaterally (NnS1 and NnS3), its insertion aponeurosis also inserted onto TBm. In one case (NnS4‐RTL), the RN branch to TFA (RN^TFA^) originated independently and proximally to the branch to TBLo (Figure 4).

**FIGURE 4:**
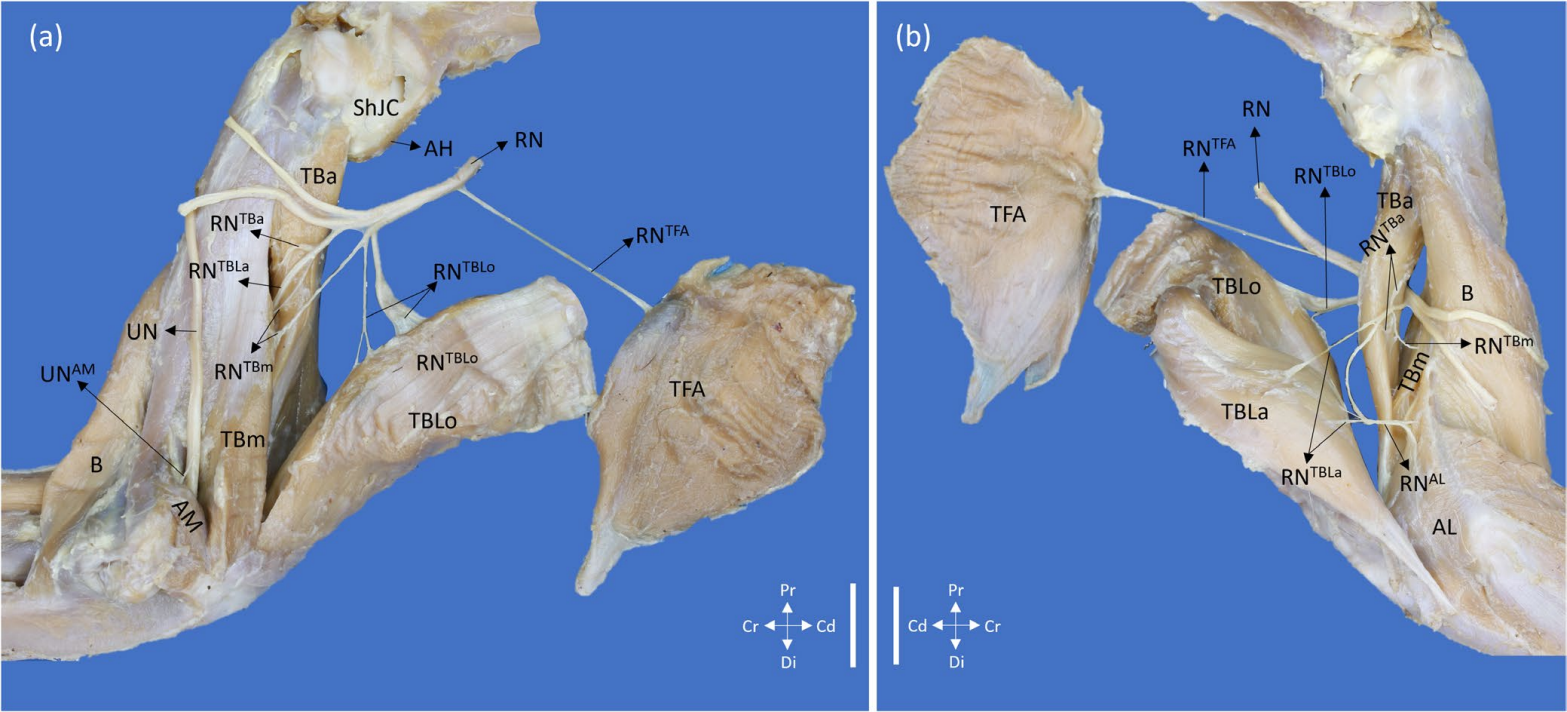
Distribution of the radial and ulnar nerves to the caudal brachial muscles in a right brachium of *Nasua nasua* . (a) Medial view; (b) lateral view. AH, *m. articularis humeri*; AL, *m. anconeus lateralis*; AM, *m. anconeus medialis*; B, *m. brachialis*; Cd, caudal; Cr, cranial; Di, distal; Pr, proximal; RN, radial nerve (*n. radialis*); RN^AL^, branch of RN to AL; RNdp, deep branch of RN (*n. radialis ramus profundus*); RN^TBLa^ branch of RN to TBLa; RN^TBLo^, branch of RN to TBLo; RN^TBm^, branch of RN to TBm; RN^TFA^, branch of RN to TFA; RN^TFA‐TBLo^; ShJC, shoulder joint capsule; TB, *m. triceps brachii*; TBa, *caput accessorium* of TB; TBLa, *caput laterale* of TB; TBLo, *caput longum* of TB; TBm, *caput mediale* of TB; TFA, *m. tensor fasciae antebrachii*; UN, ulnar nerve; UN^AM^, branch of UN to AM. Scale bars of 10 mm.

#### 3.2.2 *m. triceps brachii* (TB)

The TB was a multipennate muscle with four heads: *caput longum* (TBLo), *caput laterale* (TBLa), *caput accessorium* (TBa) and *caput mediale* (TBm) (Figures 1, 2 and 5). The common branch of RN to TFA and TBLo (RN^TFA‐TBLo^) gave rise to two branches to innervate the TBLo (RN^TBLo^), proximal and distal. The proximal branch was relatively greater than the distal branch (Figure 4). In one case (NnS4‐RTL), the proximal innervating branch originated independently to that to TFA (Figure 3). The RN gave rise to a branch to TBm distal to RN^TFA‐TBLo^ (Figures 3 and 4), and more distally to a branch that branched to TBa and TBm during its passage between both heads. The branch continued to TBLa and ended in the *m. anconeus lateralis* (AL) (Figure 4). In two cases (NnS2‐RTL and NnS7‐RTL), the RN^TBa^ perforated TBa and continued to the TBLa (as shown in Figure S2b). The arterial supply to TB was by the caudal circumflex humeral artery (CdCHA), CRA, DBA and collateral ulnar artery (CUA). The CdCHA passed proximally between the TBa and TBLo, supplying both heads and TBLa. This artery gave rise to a descending branch, the CRA, which passed between TBa and TBLa, supplied both heads and TBLo (Figure 3, Table 2). The DBA originated proximally from BA, distal to the common tendon of TMaj and LD, and supplied TB heads, except TBLa. The CUA originated distally from BA, proximal to the supracondylar foramen, and supplied TBm and *m. anconeus medialis* (AM). In one case (NnS2‐RTL), the distal bicipital artery additionally supplied TBm and in another case by the nutrient humeral artery (NnS4‐RTL).

**FIGURE 5:**
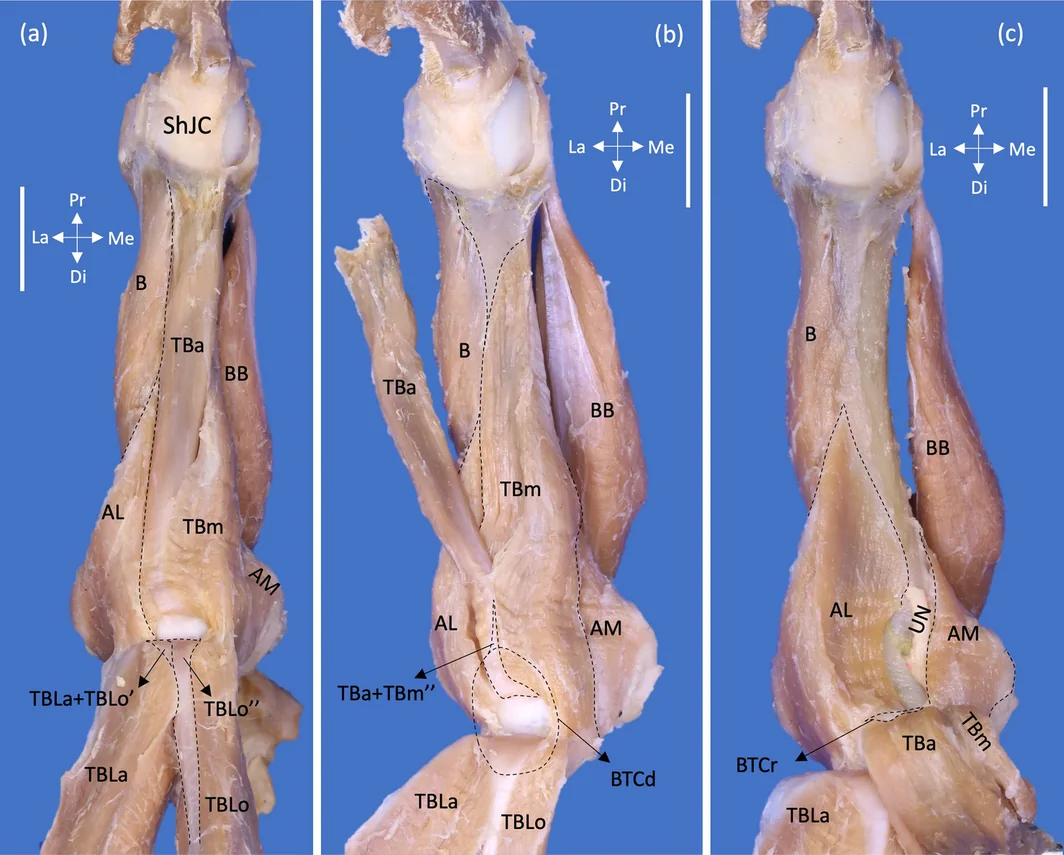
Deep caudal views of muscles of a left brachium in *Nasua nasua* . (a) The *caput laterale* (TBLa) and *caput longum* (TBLo) of *m. triceps brachii* were displaced caudally. (b) The *caput accessorium* of *m. triceps brachii* (Tba) was displaced laterally. (c) The *caput mediale* (TBm) and TBa were displaced caudally. AL, *m. anconeus lateralis*; AM, *m. anconeus medialis*; B, *m. brachialis*; BB, *m. biceps brachii*; BTCd, *bursa tricipitalis caudalis* (Caudal tricipital bursa); BTCr, *bursa tricipitalis cranialis* (Cranial tricipital bursa); Di, distal; La, lateral; Me, medial; Pr, proximal; ShJC, Shoulder joint capsule; TB, *m. triceps brachii*; TBa + TBm”, common insertion tendon of TBa and TBm; TBLa+TBLo”, common insertion tendon of TBLo and TBLa; TBLo”, insertion tendon of TBLo; UN, ulnar nerve. Scale bars: 10 mm.

##### 3.2.2.1 *m. triceps brachii caput longum* (TBLo)

The TBLo was a bipennate head that originated via a strong tendon from the ventral third of the caudal margin of scapula and infraglenoid tubercle. Inside the origin tendon, fleshy fibres also originated directly from the caudal margin of scapula. The TBLo inserted onto the caudal surface of the olecranon tuberosity via a tendon that also fused medially and caudally to the TFA tendon and laterally to the TBLa tendon (Figure 2). The lateral aspect of the TBLo belly received fleshy fibres from TBLa at the distal third of the brachium (Figure 5).

##### 3.2.2.2 *m. triceps brachii caput laterale* (TBLa)

The TBLa was a unipennate head that originated via an aponeurosis from the lateral aspect of the humeral neck (proximal to the origin of B), tricipital line and brachial fascia over B (Figures 1 and 2). The TBLa sent fleshy fibres to the TBLo belly at the distal third of the brachium, and tendon fibres more distally, forming a common tendon with TBLo that inserted onto the caudolateral aspect of the olecranon tuberosity (Figures 2 and 5). The tendon of TBLa laterally took the shape of an aponeurosis, which covered AL and inserted independently onto the caudolateral margin of the olecranon (Figure 2). As an anatomical variant (Table 3), in one case (NnS7‐RTL), the TBLa partially fused to TBa and only formed a small space for the passage of CRA (as shown in Figure S2b). In that same case (NnS7‐RTL), a lateral accessory belly originated from the proximal extreme of the lateral supracondylar crest and sent fleshy fibres to TBLa and AL (see Figure S2b). The branch of RN to TBLa innervated this accessory belly and perforated it to continue to AL.

##### 3.2.2.3 *m. triceps brachii caput accessorium* (TBa)

The TBa was a bipennate head that originated mainly via fleshy fibres from along the humeral neck and proximal fourth of the caudal surface of the humeral shaft. It also originated via tendinous fibres in the lateral and medial aspects of its belly (Figures 1 and 2). It received fleshy fibres from TBm and inserted via a common tendon onto the cranial aspect of the olecranon tuberosity (Figures 2 and 5).

##### 3.2.2.4 *m. triceps brachii caput mediale* (TBm)

The TBm was a unipennate head that originated from the medial aspect of the humerus in the zone between the origin of TBa and proximal to the supracondylar foramen (Figures 1 and 2). It originated via tendinous fibres in its proximal third and via fleshy fibres from its distal third. It also originated via fleshy fibres from the second quarter of the caudal aspect of the humerus in a zone located between TBa and proximal extreme to the *m. anconeus lateralis* (AL) (Figures 5 and 6). The proximal fleshy fibres of its belly joined to the TBa tendon of insertion and inserted via a common tendon onto the cranial aspect of the olecranon tuberosity. The distal fleshy fibres of the TBm belly inserted directly onto the medial aspect of the olecranon tuberosity (Figure 2). Two anatomical variants were observed (Table 3): In one case (NnS2‐LTL), some fibres of its origin fused to TBa, although the passage of RN separated them. In two cases (NnS3‐LTL, NnS6‐LTL), the TBm did not originate proximal to the supracondylar foramen but more proximally, leaving a space free of origin between the humeral shaft and the supracondylar foramen.

**FIGURE 6:**
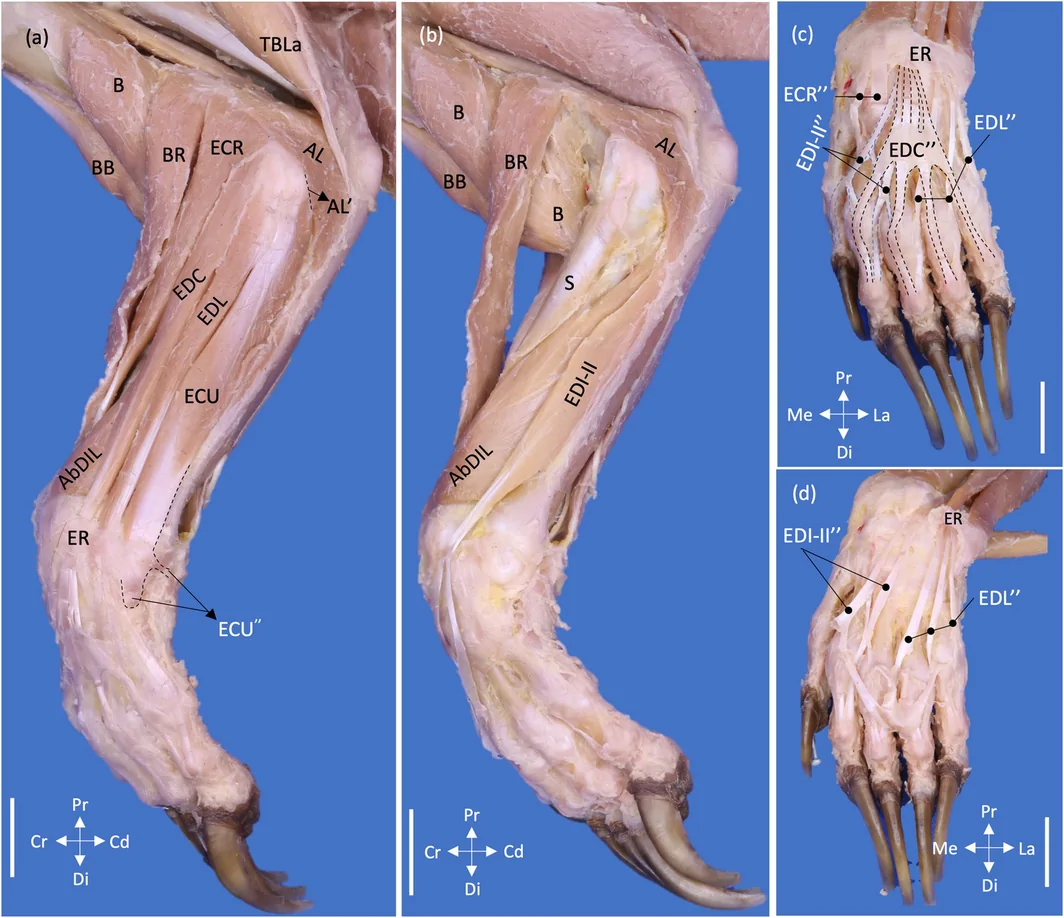
Craniolateral antebrachium muscles of *Nasua nasua* , left thoracic limb. (a) Superficial lateral view; (b) deep lateral view; (c) superficial dorsal view of manus; (d) deep dorsal view of manus. AbDIL, *m. abductor digiti I longus*; AbDIL”, insertion tendon of AbDIL; AL, *m. anconeus lateralis*; AL’, origin of AL from the *m. extensor carpi ulnaris* (ECU); B, *m. brachialis*; BB, *m. biceps brachii*; BR, *m. brachioradialis*; Cd, caudal; Cr, cranial; Di, distal; ECR, *m. extensor carpi radialis*; ECR”, insertion tendons of ECR; ECU, *m. extensor carpi ulnaris*; ECU”, insertion tendons of ECU; EDC, *m. extensor digitorum communis*; EDC”, insertion tendons of EDC; EDI‐II, *m. extensor digiti I et II*; EDI‐II’, insertion tendons of EDI‐II; EDL, *m. extensor digitorum lateralis*; EDL”, insertion tendons of EDL; ER, extensor retinaculum; IMS, intermuscular septum with the caudomedial antebrachium muscles; La, lateral; Me, medial; Pr, proximal; S, *m. supinator*; TBLa, *caput laterale* of *m. triceps brachii*. Scale bars: 10 mm.

##### 3.2.2.5 Tricipital Bursae

There were two tricipital bursae between the tendons of insertion that other authors did not report (Table 2). One cranial tricipital bursa (BTCr) was located between the AL and common tendon of TBa and TBm (see Figure S3c). The caudal tricipital bursa (BTCd) was located between the common tendon of TBLo and TBLa and the common tendon of TBa and TBm (Figures 2 and 5; Figure S3a,b).

#### 3.2.3 *m. anconeus lateralis* (AL)

The AL (‘*m. anconeus*’ of NAV) was a pyramidal muscle that originated via fleshy fibres from the distal half of humerus, including the caudal aspects of lateral supracondylar crest and lateral epicondyle. Its origin extended almost along the proximal surface to the olecranon fossa, reaching the caudoproximal surface to the supracondylar foramen (Figures 1, 2 and 3). It also originated from the origin tendon of *m. extensor carpi ulnaris* (ECU) (Figure 6). It inserted via fleshy fibres onto the cranial and lateral aspects of olecranon (Figure 2). The distal extreme of its insertion was parallel to the lateral coronoid process of ulna, covering a small portion of the *m. extensor digiti I et II* (EDI‐II). The insertion was tendinous onto the proximolateral surface of olecranon tuberosity and the belly was fused deeply with the lateral aspect of elbow joint capsule. The RN branch to AL first innervated the TBLa (Figure 4). The CUA, CRA (Figure 3), and recurrent cranial interosseous arteries supplied it. As anatomical variants (Table 3), in one case (NnS3‐LTL), the AL additionally inserted via tendinous fibres onto insertion tendon of TBa, and in another case onto the common tendon of TBLa and TBLo (NnS1‐LTL).

#### 3.2.4 *m. anconeus medialis* (AM)

The AM was a triangular muscle that originated via fleshy fibres from the proximomedial margin of the supracondylar foramen and the medial epicondyle of the humerus. It inserted mainly via fleshy fibres onto the medial aspect and distal margin of a process formed medially on the olecranon tuberosity (MPOT ‐medial process of olecranon tuberosity‐ as shown in Figure 2), and a few tendinous fibres onto the proximal extreme of the olecranon tuberosity (Figures 1, 2 and 5). The ulnar nerve (UN) innervated it (Figure 4; Figure S2), and the CUA supplied it (Figure 3).

### 3.3 Craniolateral Muscles of Antebrachium

#### 3.3.1 *m. brachioradialis* (BR)

The BR was a fusiform muscle that originated via fleshy fibres from almost the proximal half of lateral supracondylar crest, except for a short proximal portion where B originated (Figure 2). Superficially, a few tendon fibres were observed in its origin, and its muscle belly extended along the antebrachium. It inserted via a short tendon onto the proximal and cranial surface to the radial styloid process (just proximal to the groove of radius to the tendon of *m. abductor digiti I longus* [AbDIL]) (Figures 6 and 7, Table 4). The deep branch of RN (RNdb), RCA, TCA, and radial artery (RA) supplied it (Figure 8). As anatomical variants (Table 3), in two cases (NnS2‐RTL and NnS4‐LTL), it shared fibres with ECR, and in one of those cases (NnS2‐RTL), fleshy fibres of B extended to belly of BR, and between them passed the RN^db^ (as shown in Figure S4).

**FIGURE 7:**
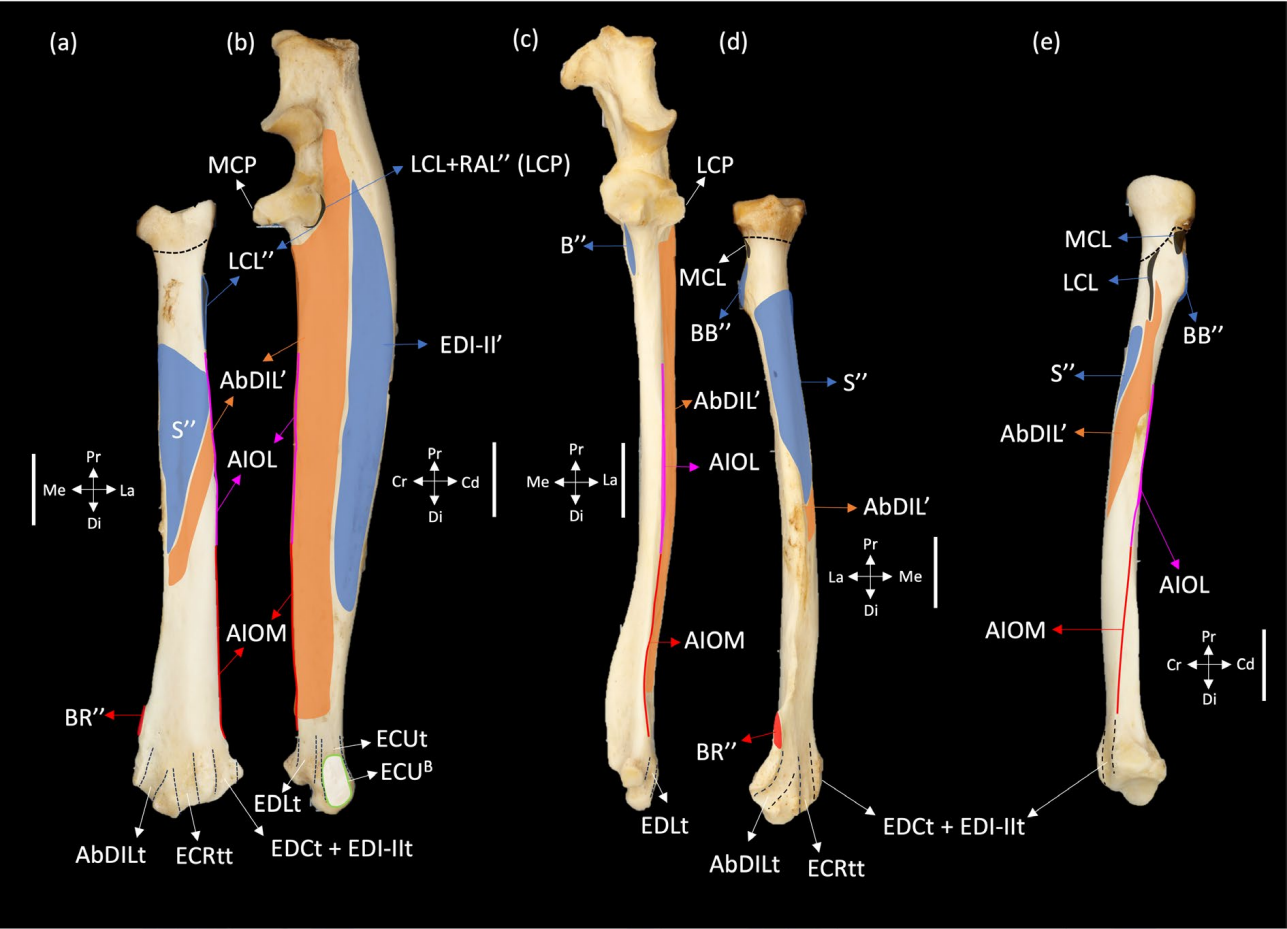
Muscle mapping of *Nasua nasua* , left radius and left ulna. (a) Cranial view of radius; (b) lateral view of ulna; (c) cranial vie of ulna; (d) medial view of radius; (e) lateral view of radius. AbDIL’, origin of *m. abductor digiti I longus*; AbDILt, groove of insertion tendon of *m. abductor digiti I longus*; AIOL, antebrachial interosseus ligament; AIOM, antebrachial interosseus membrane; B”; insertion of *m. brachialis*; BB”, insertion of *m. biceps brachii*; BR”, insertion of *m. brachioradialis*; Cd, caudal; Cr, cranial; Di, distal; ECRtt, groove of tendons of *m. extensor carpi radialis*; ECUt, groove of tendon of *m. extensor carpi ulnaris*; ECU^B^, synovial bursa of ECU; EDCt+EDI‐IIt, groove of tendons of *m. extensor digitorum communis* and *m. extensor digiti I et II*; EDI‐II’, origin of *m. extensor digiti I et II*; EDLt, groove of tendons of *m. extensor digitorum lateralis*; La, lateral; LCL”, insertions of lateral collateral ligament of elbow; LCL + RAL”(LCP), common insertion of LCL and RAL; LCP, lateral coronoid process of ulna; MCP, medial coronoid process of ulna; Me, medial; Pr, proximal; RAL, radial annular ligament; S″, insertion of *m. supinator*. Scale bars: 10 mm.

**TABLE 4:** Summary of characteristics of craniolateral antebrachium muscles in *Nasua nasua* , and main differences between the present study and other studies in the genus *Nasua*.

Muscle (abbreviation)	Shape	Origin	Insertion	Innervation and arterial supply	Differences with other studies
*m. brachioradialis* (BR)	Fusiform	Lateral supracondylar crest of humerus	Proximal and cranial surface to the radial styloid process	Deep branch of radial nerve, transverse cubital, collateral radial and radial arteries.	Sharing fibres in the origin with ECR (Tarquini et al. 2023)
		Variant: Shared fibres with *m. extensor carpi radialis* (two cases)			All cases (Tarquini et al. 2023)
*m. extensor carpi radialis* (ECR)	Bipennate (It distally forms two unipennate bellies)	Lateral supracondylar crest of humerus	Bases of metacarpal bones II and III	Deep branch of radial nerve	Two *extensor carpi radialis* muscles (longus and brevis) (McClearn 1985; Tarquini et al. 2023)
					Shared fibres with EDC and AbDIL (Tarquini et al. 2023)
*m. extensor digitorum communis* (EDC)	Bipennate	Lateral supracondylar crest, lateral epicondyle and adjacent intermuscular septa with ECR and EDL	Bases of distal phalanges of digits II–V	Deep branch of radial nerve and cranial interosseus artery	Tarquini et al. (2023) observed two bellies in two specimens and shared fibres with EDL.
					Unique origin from the lateral epicondyle (Santos, Bertassoli, Oliveira, et al. 2010; Tarquini et al. 2023)
		Origin tendon in its deep aspect in all cases			Unique origin from the lateral supracondylar crest (McClearn 1985)
		Variant: origin from joint capsule of elbow (one case)			Origin tendon only present in some cases (Tarquini et al. 2023)
					Variant tendon to digit I in one case (Tarquini et al. 2023)
*m. extensor digitorum lateralis* (EDL)	Multipennate (Two bipennate bellies, lateral and medial)	Lateral epicondyle of humerus, lateral collateral ligament of elbow and intermuscular septum with ECU	Insertion tendons of EDC to digits III–V	Deep branch of radial nerve and cranial interosseus artery	Unique origin from the lateral epicondyle (Santos, Bertassoli, Oliveira, et al. 2010; Tarquini et al. 2023).
	Lateral belly to digit V and medial belly to digit III and IV				Lateral belly to digit IV and V and medial belly to digit III (Tarquini et al. 2023)
*m. extensor carpi ulnaris* (ECU)	Bipennate	Lateral epicondyle of humerus and fusion with the joint capsule of elbow	Lateral aspect of the base of metacarpal bone V and accessory carpal bone	Deep branch of radial nerve and cranial interosseus artery	No fusion with joint capsule of elbow
					Additional origin from the olecranon (Mackintosh 1875)
					No insertion onto the carpal accessory bone (Mackintosh 1875; McClearn 1985; Meckel 1829; Tarquini et al. 2023).
*m. supinator* (S)	Fusiform	Lateral epicondyle of humerus, lateral collateral ligament of elbow and radial annular ligament	Proximal half of cranial surface and medial margin of shaft of radius	Deep branch of radial nerve	No origin from radial annular ligament (Tarquini et al. 2023)
					Origin from the joint capsule of elbow (Tarquini et al. 2023)
					Unique origin from the lateral epicondyle of humerus (Santos, Bertassoli, Oliveira, et al. 2010)
					Insertion onto the middle third in *Nasua* (Mackintosh 1875; Tarquini et al. 2023)
					Muscle no reported (McClearn 1985)
*m. abductor digiti I longus* (AbDIL)	Bipennate	Proximal half of medial margin of radius and almost the entire lateral surface of ulna	Sesamoid bone of AbDIL and metacarpal bone I	Deep branch of radial nerve, caudal and cranial interossei arteries	No origin from radius (Mackintosh 1875; Santos, Bertassoli, Oliveira, et al. 2010)
					No origin from antebrachial interosseous membrane and ligament
					Unique insertion onto the metacarpal bone I (McClearn 1985; Santos, Bertassoli, Oliveira, et al. 2010)
					Unique insertion onto the sesamoid bone (Mackintosh 1875)
					Distinct distribution of origin zones in the muscle maps of radius and ulna (Tarquini et al. 2023)
*m. extensor digiti I et II* (EDI‐II)	Bipennate	Lateral surface of ulna, caudal to the AbDIL	Tendons of EDC to digits I‐III	Deep branch of radial nerve and cranial interosseus artery	
		Variant: Distal to the lateral coronoid process.			Typical pattern (Tarquini et al. 2023)
					No tendon to digit III (Böhmer et al. 2020; Mackintosh 1875; Meckel 1829; Tarquini et al. 2023; Windle 1888).
			Insertion tendons pass by the same compartment of EDC tendons		Tendons pass by lateral groove of radius (Tarquini et al. 2023)
					Muscle no reported (Santos, Bertassoli, Oliveira, et al. 2010)

**FIGURE 8:**
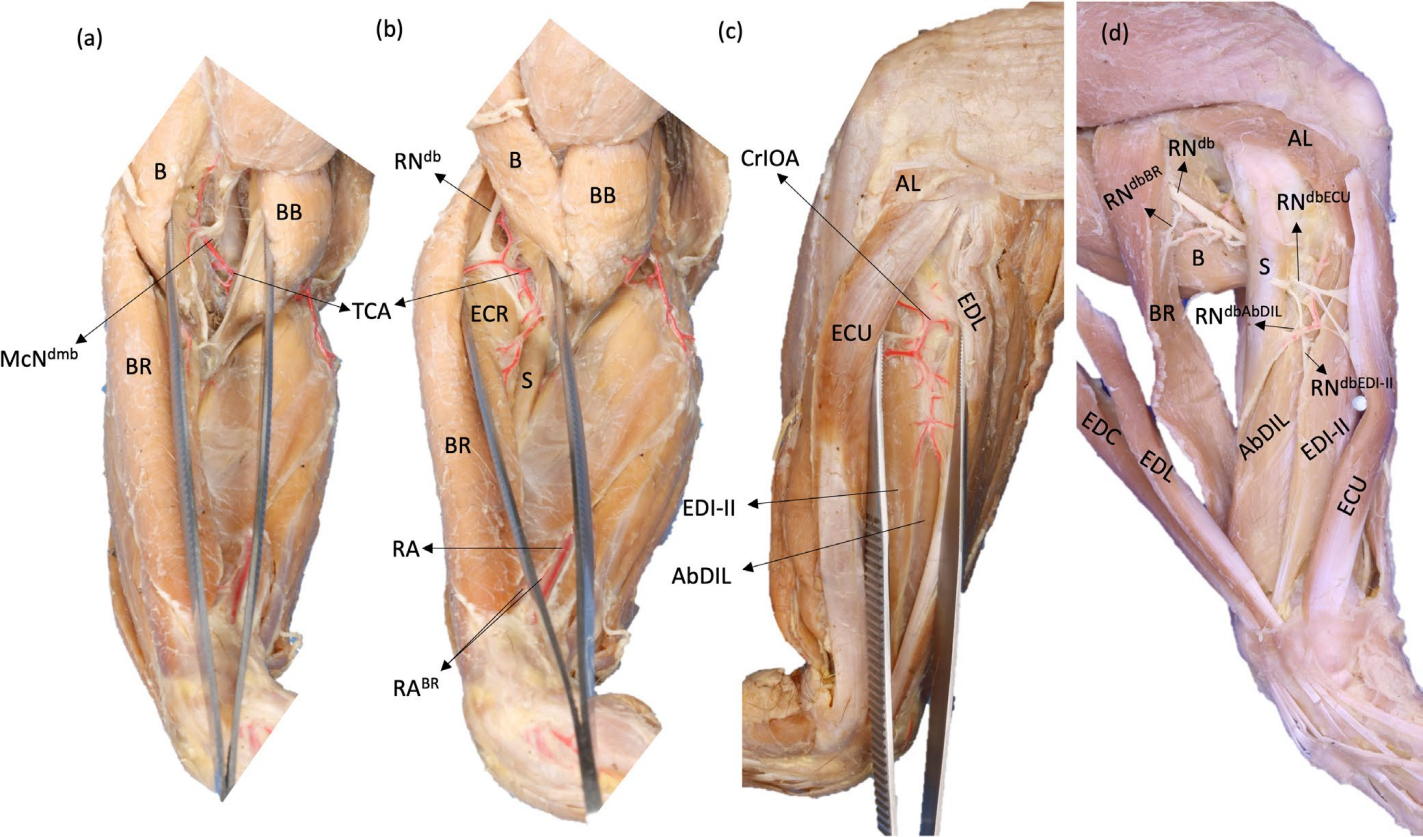
Arterial and nerve supply of the craniolateral antebrachial muscles of *Nasua nasua* . (a) Craniomedial view of a right thoracic limb separating between B and BB. (b) Craniomedial view of a right thoracic limb separating between the B and BR. (c) Lateral view separating between the EDL and ECU. (d) Lateral view of a left thoracic limb after remove the ECR, EDC and EDL. CrIOA, cranial interosseus artery; McN^dmb^, distal muscular branch of musculocutaneous nerve; RA, radial artery; RA^BR^, branch of RA to BR; RN^db^, Deep branch of the radial nerve; RN^dbAbDIL^, branch of RN to AbDIL; RN^dbBR^, branch of RN to BR; RN^dbEDI‐II^, branch of RN to EDI‐II; TCA, transverse cubital artery.

#### 3.3.2 *m. extensor carpi radialis* (ECR)

The ECR was an undivided muscle at the proximal third of antebrachium and only from the middle third divided into two unipennate bellies (see Figure S5), cranial and caudal, which correspond to *m. extensor carpi radialis longus* (ECRL) and *m. extensor carpi radialis brevis* (ECRB), respectively. It originated via fleshy fibres from the distal two‐sixths of lateral supracondylar crest (distal to BR) and the intermuscular septum with *m. extensor digitorum communis* (EDC) (Figure 6). In its deep aspect, it also originated via a common tendon with EDC and via fleshy fibres medial to that tendon. Part of its origin was distally covered by the origin of EDC (Figure 2). Both bellies of ECR extended until the second third of antebrachium, although the caudal belly extended a few more than the cranial belly. Its tendons were free of fleshy fibres just proximal to their deep passage to the AbDIL tendon and continued distally through a groove of radius formed lateral to the groove of AbDIL (Figure 7). The cranial belly inserted via a tendon along the dorsal aspect of base of metacarpal bone II. The caudal belly inserted via a tendon onto the dorsomedial aspect of base of metacarpal bone III. The RN^db^ and TCA supplied it (Figure 8; Table 4; Figure S5).

#### 3.3.3 *m. extensor digitorum communis* (EDC)

The EDC was a bipennate muscle that originated via fleshy fibres from the distal extreme of the lateral supracondylar crest, lateral epicondyle and adjacent intermuscular septa with ECR and *m. extensor digitorum lateralis* (EDL) (Figures 2 and 7). The origin was also via a tendon in its deep aspect. The fleshy belly extended until the proximal four‐fifths of the antebrachium. The belly formed four tendons that pass through the superficial compartment of extensor retinaculum and a groove of radius (lateral to ECR groove, Figure 7). These tendons formed an aponeurosis at the middle level of metacarpal region and distally separated again for digits II–V (Figure 6). The tendons fused with the joint capsules of proximal interphalangeal joints and continued distally between the elastic dorsal ligaments toward the dorsal aspect of bases of distal phalanges of digits II–V. The RNdb and cranial interosseous artery (CrIOA) supplied it (Figure 8; Table 4). As anatomical variants (Table 3), it additionally originated from the joint capsule of elbow in one case (NnS2‐RTL), and in another case (NnS5‐RTL), the belly only formed three tendons where the most medial tendon formed the tendons to digits II and III (as shown in Figure S6).

#### 3.3.4 *m. extensor digitorum lateralis* (EDL)

The EDL was a multipennate muscle that originated via a tendon from the lateral epicondyle of the humerus and via fleshy fibres from the lateral collateral ligament of the elbow and intermuscular septum formed by the antebrachial fascia to separate it from ECU (Figures 2 and 6). It formed two bellies, one lateral that extended to digit V and one medial to digits III and IV (Figure 6). The lateral belly was smaller and formed its tendon more proximally than the medial belly. The fleshy belly of the medial belly was observed passing deep to the extensor retinaculum. The tendon to digit III also sent a weak fascicle to the axial aspect of digit IV, which inserted onto the metacarpophalangeal joint and base of the proximal phalanx. The RNdb and CrIOA supplied it (Figure 8; Table 4).

#### 3.3.5 *m. extensor carpi ulnaris* (ECU)

The ECU was a bipennate muscle that originated from the lateral epicondyle of humerus via a tendon (Figures 2 and 6), which fused deeply with the joint capsule of elbow (as shown in Figure S7). It inserted via a tendon onto the lateral aspect of base of metacarpal bone V. There was a synovial bursa between the tendon and the ulnar styloid process (as shown in Figure S7). Its tendon fused with the retinaculum extensor and sent a small tendinous fascicle to the accessory carpal bone (Figure 6). The RNdb and CrIOA supplied it (Figure 8; Table 4).

#### 3.3.6 *m. supinator* (S)

The S was a fusiform muscle that originated via a tendon from the lateral collateral ligament of elbow and lateral epicondyle of humerus (deep to the lateral collateral ligament) (Figure 2), and via fleshy fibres from the radial annular ligament. It inserted via fleshy fibres onto the proximal half of cranial surface and medial margin of shaft of radius (at the level just distal to radial tuberosity) (Figures 6 and 7). The RNdb and CrIOA supplied it (Figure 8; Table 4).

#### 3.3.7 *m. abductor digiti I longus* (AbDIL)

The AbDIL was a bipennate muscle that originated via fleshy fibres from the proximal half of the medial margin of radius and almost from the entire lateral surface of ulna (Figure 7). The extreme caudal of its origin is via tendinous fibres (as shown in Figure S8a). At the level of radius, it covered the zone distal to the insertion of the lateral collateral ligament of elbow (Figures 6 and 7), antebrachial interosseous ligament, and proximal half of antebrachial interosseous membrane (as shown in Figure S8b). At the level of the ulna, it covered the zone parallel to the anconeus process until the proximal zone to the ulnar styloid process (Figure 7). It inserted via a tendon onto the sesamoid bone and mediopalmar aspect of the base of metacarpal bone I. The RNdb, CrIOA (Figure 8) and caudal interosseous artery (CdIOA) supplied it (as shown in Figure S8c) (Table 4). As an anatomical variant (Table 3), it also originated from the radial fascicle of the lateral collateral ligament of elbow in one specimen bilaterally (NnS4).

#### 3.3.8 *m. extensor digiti I et II* (EDI‐II)

The EDI‐II was a bipennate muscle that originated via fleshy fibres from the lateral surface of the ulna, caudal to the AbDIL (Figures 6 and 7). Its origin began parallel to the distal half of the lateral coronoid process of the ulna where it was covered by AL. Some fleshy fibres also originated from the caudal extremity of the AbDIL (as shown in Figure S8a). The EDI‐II formed two tendons, medial and lateral, which extended to the digits I–III. The medial tendon only extended to digit I and inserted onto the base of its distal phalanx. The lateral tendon divided into two tendons to the abaxial and axial aspects of digit II. The axial tendon sent a fascicle to digit III (Figure 6). These tendons passed deep to the tendons of EDC through the same compartment of the extensor retinaculum and radial groove. These tendons joined to the EDC tendon at the distal extreme of the dorsal aspect of proximal phalanges (Figure 6). The RNdb and CrIOA supplied it (Figure 8; Table 4). As an anatomical variant, in one case bilaterally (NnS2), the muscle originated distal to the lateral coronoid process, and AL did not cover it (Table 3).

## 4 Discussion

Muscle homology and evolutionary derivation inferences were based on the criteria outlined by Diogo et al. (2018), including evidence from dissections and available literature, relationships with bones and adjacent muscles, fibre orientation, innervation, function, absence or presence of muscles and embryological development. This latter was considering the most recent study of embryological splitting of dorsal and ventral derivations of thoracic limb musculature in amniotes (Smith‐Paredes et al. 2022). To trace the evolutionary line of mammals leading to *Nasua nasua* , our inferences also relied on comparative analyses of anatomical descriptions and muscle maps of monotremes, marsupials and representative terrestrial members of Scrotifera (Granorder Scrotifera, following the phylogenetic framework of Christmas et al. 2023), including cetartiodactyls, perissodactyls, pangolins and carnivorans of the suborder Caniformia.

### 4.1 Dorsal Musculature: Triceps Subdivision

#### 4.1.1 *m. tensor fasciae antebrachii* (TFA)

In *N. nasua* , the TFA was an undivided muscle, corroborating previous descriptions (Böhmer et al. 2020; Mackintosh 1875; Santos, Bertassoli, Oliveira, et al. 2010; Tarquini et al. 2023). Notably, we found it originating also from the common tendon of several extrinsic thoracic limb muscles (cutaneus trunci, latissimus dorsi, pectoralis profundus and pectoralis abdominalis), which is the same origin of the cranial part of TFA (TFA *pars cranialis*) in other procyonids (Feeney 1999; Vélez García et al. 2024; Vélez‐García, Carrión Blanco, et al. 2023). Besides, the arrangement of its belly in the medial aspect of the brachium is like whether the cranial and caudal parts were fused in other procyonids (Figure 9) (Vélez García et al. 2024; Vélez‐García, Carrión Blanco, et al. 2023). Thereby, the *pars cranialis* of TFA evolutionarily might not have been lost but rather fused with the *pars caudalis* in *N. nasua* . This could have occurred during the divergence of the genus *Nasua* within the family Procyonidae (Table 6). The division of this muscle has also been described in several representatives of the superfamily Arctoidea with a similar arrangement to that described in procyonids different from *N. nasua* (Vélez‐García, Carrión Blanco, et al. 2023), thus the common ancestor of arctoids could potentially have had a divided TFA.

**FIGURE 9:**
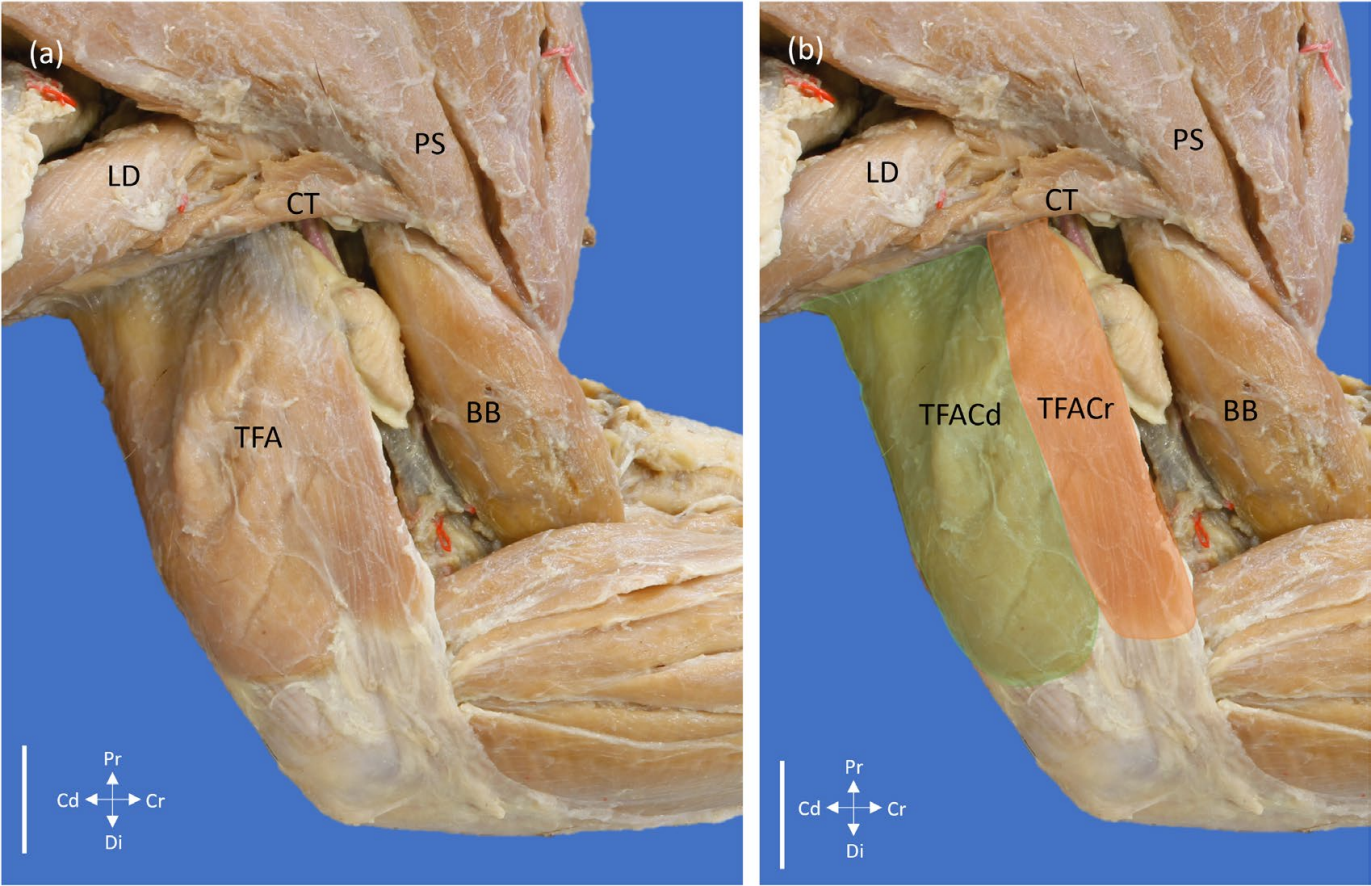
Homologous cranial and caudal parts of tensor fasciae antebrachii muscle of *Nasua nasua* to other procyonids. (a) Figure 1d of the present study, (b) same photography of with the homologous parts of TFA of other procyonids in green and orange. CT, *m. cutaneus trunci*; LD, *m. latissimus dorsi*; PS, *mm. pectorales superficiales*; PP, *m. pectoralis profundus*; TFA, *m. tensor fasciae antebrachii*; TFACr, homologous part of TFA *pars cranialis* of other procyonids (orange); TFACd, homologous part of TFA *pars caudalis* of other procyonids TFA (green). Scale bars: 10 mm.

The innervation to TFA from a branch of radial nerve (RN) that also extends to TBLo, and the fusion with TBLo tendon in *N. nasua* allows us to hypothesize that TFA is derived from TBLo, as is suggested in other procyonids (Vélez García et al. 2024). Diogo et al. (2018) hypothesized that TFA could be homologous to the triceps coracoideus of Reptilia, while Smith‐Paredes et al. (2022) found that TFA is not homologous to some of the TB heads of reptiles. It is an exclusive mammalian muscle derived from the triceps division (Smith‐Paredes et al. 2022) (Table 6).

#### 4.1.2 *m. triceps brachii* (TB)

The TB of *N. nasua* consists of only four heads as was determined in most caniforms (Vélez‐García, Carrión Blanco, et al. 2023). The AM is not a head of TB in *N. nasua* , in agreement with studies performed in other procyonids (Vélez García et al. 2024; Vélez‐García, Carrión Blanco, et al. 2023) and with evidence from mammalian embryological development (Diogo et al. 2018; Smith‐Paredes et al. 2022). This observation contrasts with previous studies in which AM was considered an additional head of TB (Böhmer et al. 2020; Tarquini et al. 2023).

Some authors have controversially excluded the TBa in carnivorans, and in their muscle maps, depicting TBm in their muscle maps as covering the origin zone of TBa (Böhmer et al. 2020; English 1977) (Table 4). By contrast, several studies have confirmed the presence of TBa in many of the same species examined by those authors (Feeney 1999; Leach 1977; Tarquini et al. 2023; Vélez‐García, Carrión Blanco, et al. 2023). In *N. nasua* , our review of the radial nerve (RN) distribution revealed that one branch to TBLa first gave rise to a branch to TBa, and in some cases perforated TBa before innervating TBLa. Additionally, partial fusion of TBLa with TBa was observed in one specimen of *N. nasua* . This condition was also reported as an anatomical variant in the crab‐eating raccoon ( *Procyon cancrivorus* ) (Vélez García et al. 2024) and within the typical pattern of mustelids of the genus *Martes* (Hall 1926; Leach 1977).

The TBa is present in early‐diverging mammalian groups such as monotremes (Gambaryan et al. 2015; Diogo et al. 2016; Harvey and Warburton 2010; Richards et al. 2023; Stein 1981; Warburton and Marchal 2017) and most non‐carnivoran terrestrial scrotifers (Barone 2020a; Etienne et al. 2021; Fisher et al. 2007; Humphry 1869; Kawashima et al. 2015; MacLaren and McHorse 2020). Some terrestrial scrotifers have TBa, such as domestic small ruminants (*Capra aegagrus hircus* and *Ovis aries* ) (Barone 2020a; Kamali 2025c), wild small ruminants (Mazama spp. and *Subulo gouazoubira*) (Stipp et al. 2026) and domestic pig ( *Sus scrofa* ) (Barone 2020a). Based on the anatomical descriptions and muscle maps of species lacking TBa, the TBLa appears to cover the origin area of TBa in monotremes and scrotifers (small ruminants, pigs and carnivorans). For this reason, we infer that TBa may be derived from TBLa in scrotifers, a hypothesis supported by our findings on topology and innervation in *N. nasua* as well as by comparative data from other species.

In the mustelid lesser grison ( *Galictis cuja* ), the TBm is divided into three heads (Ercoli et al. 2015). Among those heads, the ‘*m. triceps brachii caput mediale principal*’ occupies the medial aspect of the origin zone of TBa, as represented in the muscle maps of other caniforms (Barone 2020b; English 1977; Hermanson et al. 2020; Howard 1973; Leach 1977; da Souza‐Junior et al. 2018; Tarquini et al. 2023; Vélez García et al. 2024; Vélez‐García, Carrión Blanco, et al. 2023). Subsequently, the ‘*m. triceps brachii caput mediale principal*’ of *G. cuja* described by Ercoli et al. (2015) may represent a derivation from TBa rather than from TBm. This interpretation is supported by a recent study in one case of domestic dog ( *Canis lupus familiaris* ), where the presence of an additional TB head was probably derived from TBa (Kamali 2025a). Nevertheless, a complementary derivation from TBm cannot be entirely excluded because the RN distribution has not been studied in detail in several caniforms. Moreover, the TBa seems to be completely fused with TBm in some marsupials (Argot 2001; Richards et al. 2023). Nonetheless, examination of figures from recent brachial plexus studies in marsupials (Bakıcı et al. 2024; Natsuyama et al. 2024) reveals that the RN passes through the *m. triceps brachii* at the same level as in monotremes (Gambaryan et al. 2015) and scrotifers (Barone and Simoes 2010; Humphry 1869; Vélez‐García, de Carvalho Barros, and Miglino 2023). In reptiles, two humeral triceps brachii heads are present—the *triceps humeralis lateralis* (THLa) and *triceps humeralis medialis* (THm)—that are homologous to the TBLa and TBm of mammals, respectively (Smith‐Paredes et al. 2022). Thus, the TBa appears to represent a homoplastic feature in monotremes and certain scrotifers (small ruminants, pigs and carnivorans), whereas within Caniformia it constitutes a synapomorphy due to its consistent presence. Accordingly, our primary hypothesis is that the TBa may be derived from the TBLa, with an alternative hypothesis suggesting that part of TBa may also be derived from the TBm (Table 6).

#### 4.1.3 *mm. anconeus lateralis* (AL) and *extensor carpi ulnaris* (ECU)

The AL is innervated by a branch of the RN that first branched to the TBLa in *N. nasua* . This same arrangement has been found in other mammals; hence, formerly, its evolutionary derivation from the TB had been hypothesized by Lewis in 1989 apud Diogo et al. (2018). In contrast, according to Diogo et al. (2018) the more accepted hypothesis is that AL could derive from the ECU (*m. extensor antebrachii et carpi ulnaris*) of a common ancestor of amniotes, appearing as an independent muscle to the ECU in monotremes. However, the most recent embryological study in amniotes found that AL and ECU derive independently from the triceps division in mammals (Smith‐Paredes et al. 2022) (Table 6). This derivation could explain several anatomical arrangements of the TBLa, AL and ECU in procyonids. Among them, the origin of AL from ECU in *N. nasua* ; the variant origin of ECU from the ulna in the North American coati ( *N. narica* ) (Mackintosh 1875), crab‐eating raccoon ( *Procyon cancrivorus* ) (Vélez‐García et al. 2022) and kinkajou ( *Potos flavus* ) (Julitz 1909); and the variant presentation of an accessory belly of TBLa originated proximal to AL in *Potos flavus* (Vélez‐García, Carrión Blanco, et al. 2023), common raccoon ( *Procyon lotor* ) (Windle and Parsons 1897) and *N. nasua* (present study).

The origin of ECU from the ulna has also been reported in ursids, such as the American black bear ( *U. americanus* ) (Shepherd 1883) and giant panda ( *Ailuropoda melanoleuca* ) (Endo et al. 2003), as well as in mustelids such as the European polecat ( *Mustela putorius* ) (Alix 1876) and Neotropical river otter ( *Lontra longicaudis* ) (Rojas‐Ramírez and Vélez‐García 2025). This could represent an ancestral arrangement since it is normally present in monotremes of family Tachyglossidae (Gambaryan et al. 2015) and in some marsupials (Richards et al. 2023; Stein 1981). The origin of an accessory belly of TBLa from the lateral supracondylar crest just proximal to AL was also observed in the marsupial *Caluromys philander* (Argot 2001).

### 4.2 Dorsal Division: Extensor Subdivisions

#### 4.2.1 *m. brachialis* (B)

Differing from the description previously reported in *N. nasua* (Tarquini et al. 2023), in the present study B originated almost from the entire length of the lateral supracondylar crest, and there was a space free of origin in the humeral shaft at the level of groove of B (*sulcus m. brachialis*). Based on muscle maps represented by several authors in caniforms, there is a space free of origin between the cranial and caudal extremes of the lateral surface of humerus. This feature can be observed in the figures of the humerus of the procyonid *P. cancrivorus* (Tarquini et al. 2023; Vélez García et al. 2024), the ursid *A. melanoleuca* (Davis 1964), the mustelid *Galictis cuja* (Ercoli et al. 2015) and the canids *C. lupus familiaris* (Barone 2020b; Hermanson et al. 2020) and African wild dog ( *Lycaon pictus* ) (Smith et al. 2020). Whilst in other caniforms, the origin of B is also represented in that space of the humerus (English 1977; Howard 1973; Leach 1977; Vélez‐García, Carrión Blanco, et al. 2023). The strong fascia connecting its belly to the elbow joint capsule in *P. cancrivorus* (Vélez García et al. 2024) was likewise present in *N. nasua* . The unique insertion onto the ulna represents the typical pattern in caniforms (Vélez‐García, Carrión Blanco, et al. 2023). Nevertheless, an additional insertion onto the radial tuberosity has been reported in *P. cancrivorus* (Pereira et al. 2010; Santos, Bertassoli, Rosa, et al. 2010) and *N. nasua* (Santos, Bertassoli, Oliveira, et al. 2010).

In monotremes, the B inserts onto both radius and ulna (Gambaryan et al. 2015), while it inserts exclusively onto the ulna in marsupials (Argot 2001; Bakıcı et al. 2024; Diogo et al. 2016; Langworthy 1932; Martin et al. 2019; Richards et al. 2023; Warburton and Marchal 2017) and pangolins (Humphry 1869; Kawashima et al. 2015). Hence, the exclusive insertion onto the ulna may represent a synapomorphy in most caniforms, while the additional insertion onto the radius constitutes an apomorphy, as observed in canids.

Diogo et al. (2018) previously hypothesized that B in reptiles derived from the *m. humeroantebrachialis* of amphibians. In contrast, Smith‐Paredes et al. (2022) found that B derived from the most cranial part of the extensor division in reptiles and mammals (Table 6), differing from Diogo et al. (2018). Consequently, the distal distribution of the musculocutaneous nerve (distal muscular branch) is not a good character to infer evolutionary derivations because B did not derive from the biceps division but from the humeral subdivision of the extensor division (Smith‐Paredes et al. 2022) (Table 6). This derivation explains why B can be accessorily innervated by RN in some scrotifers as the horse (Budras et al. 2009), ruminants (Budras and Habel 2011; Kamali 2025b) and variably some caniforms as *P. cancrivorus* (Vélez‐García, de Carvalho Barros, and Miglino 2023), *A. melanoleuca* (Davis 1964) and the ailurid red panda ( *Ailurus fulgens* ) (Carlsson 1925). In most carnivorans (including *N. nasua* ), the McN originates from the sixth and seventh cervical spinal nerves (C6 and C7) and the RN from these and the first thoracic (C6 or C7‐T1) (Vélez‐García, de Carvalho Barros, and Miglino 2023). From an embryological perspective, it is possible that the most cranial spinal roots contributing to the RN later form the distal muscular branch of the McN (McN^dmb^) during posterior embryological stages. This is because the cervical spinal nerves that give rise to the McN (C6 and C7) also contribute to the RN in most carnivorans (Vélez‐García, de Carvalho Barros, and Miglino 2023).

#### 4.2.2 *mm. brachioradialis* (BR), *extensor carpi radialis* (ECR), *extensor digitorum comunis* (EDC), *extensor digitorum lateralis* (EDL), *supinator* (S), *abductor digiti* I *longus* (AbDIL) and *extensor digiti I et II* (EDI‐II)

A study based on literature review determined that the most intra‐ and interspecific variations of the craniolateral antebrachial muscles in caniforms were the frequent absence of BR in canids, the division of ECR in ECRB and ECRL and the tendinous fascicle to digit III from EDI‐II (Vélez‐García et al. 2022). In monotremes and most marsupials there is only an ECR (Argot 2001; Gambaryan et al. 2015; Haughton 1864; Richards et al. 2023; Warburton and Marchal 2017), while in some marsupials it is divided into ECRL and ECRB (Diogo et al. 2016; Harvey and Warburton 2010; Stein 1981). This separation into ECRL and ECRB in caniforms and some marsupials is a product of homoplasy (Diogo et al. 2018; Ercoli et al. 2015).

Diogo et al. (2018) inferred that the ‘extensor digiti minimi’, ‘*extensor pollicis longus*’ and ‘*extensor indicis*’ (the former muscle homologous to EDL and the other two muscles to EDI‐II) of mammals could have derived from the dorsum of manus, similar to the *mm. extensor digitorum* breves of reptiles. However, Smith‐Paredes et al. (2022) found that these muscles derived from the extensor division with the other craniolateral antebrachial muscles (but ECU) and not from the hand extensor subdivision. Thereby, the extensor subdivision divides into radial, central and deep lobes at the level of the antebrachium (Smith‐Paredes et al. 2022). The BR and ECR (ECRL and ECRB) derive from the radial lobe, EDC and EDL from the central lobe and S, AbDIL and EDI‐II from the deep lobe. Based on the homologies established by Smith‐Paredes et al. (2022), there are no homologous muscles to S and EDII between mammals and reptiles, and S derived together with AbDIL, while EDII (*m. extensor digiti II*) derived separated to EDI (*m. extensor digiti I*) (Table 6). As a result, the fusion of both muscles as occurs in monotremes (Gambaryan et al. 2015) and caniforms could occur in later embryological stages and to be present as a single muscle. Besides this muscle may also send a tendon to digit III in the procyonids of genera *Procyon* and *Nasua* (Vélez‐García et al. 2022), as was confirmed in *N. nasua* .

### 4.3 Brachial Subdivision of Ventral Musculature

#### 4.3.1 BB

The BB in *N. nasua* has only one head, which is homologous to the *caput longum* (BBl) of other mammals that also possess a *caput breve* (BBb), such as the procyonid *P. flavus* (Vélez‐García, Carrión Blanco, et al. 2023). The presence of both BBl and BBb likely represents a plesiomorphic arrangement of the last common ancestor of amniotes (Diogo et al. 2018) and is conserved in monotremes (Gambaryan et al. 2015) and marsupials (Argot 2001; Diogo et al. 2016; Harvey and Warburton 2010; Warburton and Marchal 2017). The absence of BBb may constitute a synapomorphy in terrestrial scrotifers since it is only present in some caniforms of the infraorder Arctoidea, such as ursids of the genera *Ursus* and *Tremarctos* (Davis 1964; Kelley 1888; Shepherd 1883; Windle and Parsons 1897), the ailurid *A. fulgens* (Carlsson 1925; Davis 1964; Fisher et al. 2009) and the procyonid *P. flavus* (Beswick‐Perrin 1871; Böhmer et al. 2020; Davis 1964; Julitz 1909; Vélez‐García, Carrión Blanco, et al. 2023). Nevertheless, the BBb can be variably absent in these species (Davis 1964; Fisher et al. 2009; Vélez‐García, Carrión Blanco, et al. 2023; Windle and Parsons 1897) or variably present in *Procyon lotor* (Allen 1882). Consequently, the BBb represents a homoplastic muscle character within Caniformia, which may also have been present in the last common ancestor of procyonids. For this reason, the BBb may have been lost in extant non‐*Potos* procyonids following divergence from the genus *Potos* (Vélez‐García, Carrión Blanco, et al. 2023).

The BB, together with *mm. coracobrachiales*, derives from the brachial subdivision (Smith‐Paredes et al. 2022). According to Diogo et al. (2018) the BBl of reptiles is homologous to the *m. coracoradialis* of amphibians, and alternatively to *m. humeroantebrachialis*, while BBb is homologous to the *coracoradialis* and *coracobrachialis* muscles. Yet Smith‐Paredes et al. (2022) found that both biceps brachii heads originated from the same subdivision (Smith‐Paredes et al. 2022) (Table 6). This observation may explain why BBb occasionally appears within BBl occasionally in *P. flavus* (Vélez‐García, Carrión Blanco, et al. 2023).

Similar to our results in *N. nasua* , most studies in procyonids only observed the insertion of BB onto the radial tuberosity (Allen 1882; Beddard 1900; Beswick‐Perrin 1871; English 1977; Julitz 1909; Mackintosh 1875; Meckel 1829; Tarquini et al. 2023; Vélez García et al. 2024; Vélez‐García, Carrión Blanco, et al. 2023; Windle 1888; Windle and Parsons 1897). In contrast, some authors observed an additional insertion onto the ulnar tuberosity in the procyonids *N. nasua* , *P. cancrivorus* and *P. lotor* (Feeney 1999; Pereira et al. 2010; Santos, Bertassoli, Oliveira, et al. 2010; Santos, Bertassoli, Rosa, et al. 2010). Most caniforms only have insertion onto the radial tuberosity, but canids (Böhmer et al. 2020; Hermanson 2020; Smith et al. 2020; da Souza‐Junior et al. 2018; Vélez et al. 2018). The insertion tendon of BB can be fused to the tendon of B occasionally in the procyonids *P. cancrivorus* (Vélez García et al. 2024) and *Nasua* sp. (Meckel 1829) and the canid *L. pictus* (Smith et al. 2020). Böhmer et al. (2018, 2020) found the muscle only inserted onto the ulna in the mustelids European pine marten ( *Martes martes* ) and beech marten ( *Martes foina* ). This differs from other studies in the same genus that described the insertion only onto the radius (Hall 1926; Leach 1977; Mackintosh 1875; Meckel 1829; Yousefi et al. 2018). On this basis, the typical pattern in arctoids is the unique insertion onto the ulna.

### 4.4 Flexor Subdivisions of Ventral Musculature

#### 4.4.1 *m. anconeus medialis* (AM)

The AM is present in monotremes (Gambaryan et al. 2015), marsupials (Argot 2001; Diogo et al. 2016; Harvey and Warburton 2010; Richards et al. 2023; Stein 1981; Warburton and Marchal 2017), pholidota (Böhmer et al. 2020; Humphry 1869; Kawashima et al. 2015) and caniforms except canids and ursids (Vélez‐García, Carrión Blanco, et al. 2023). Formerly, Mackintosh (1875) described the presence of two ‘anconei’ muscles in *Nasua*, while other authors only named the presence of the AL (Meckel 1829; Santos, Bertassoli, Oliveira, et al. 2010). Although more recent authors found the AM with independent attachments to TB, they considered it as a head of TB (Böhmer et al. 2020; Tarquini et al. 2023). Thereby, Tarquini et al. (2023) named it ‘*triceps brachii caput mediale accessorium*’ and Böhmer et al. (2020) ‘triceps brachii caput accessorium’ (Table 5). In the present study, we also found the AM in all *N. nasua* specimens independent to the TB and innervated by the ulnar nerve, corroborating its presence as occurs in most caniforms (Enciso‐García and Vélez‐García 2022; Vélez García et al. 2024; Vélez‐García, Carrión Blanco, et al. 2023; Vélez‐García, de Carvalho Barros, and Miglino 2023; Windle and Parsons 1897). Even though the AM normally is not present in canids (Böhmer et al. 2019; Feeney 1999; Pereira et al. 2016; Smith et al. 2020; da Souza‐Junior et al. 2018; Vélez et al. 2018) and ursids (Meckel 1829; Shepherd 1883; Windle and Parsons 1897), it has been found vestigial in the canid crab‐eating fox ( *Cerdocyon thous* ) (Vélez‐García et al. 2018) and the ursids polar bear ( *Ursus maritimus* ) (Kelley 1888) and *A. melanoleuca* (Davis 1964). On the other hand, although in mustelids the AM is normally present, it has been observed in a vestigial form in a specimen of *G. cuja* (Ercoli et al. 2015) and not observed in a *M. putorius* (Böhmer et al. 2019).

**TABLE 5:** Comparative anatomical terminology of the brachium and craniolateral antebrachium muscles in *Nasua nasua* .

Muscle term based on NAV (2017)	Other muscle terms used by other authors	References
*m. brachialis*	*m. brachialis anticus*	Mackintosh (1875)
	*m. brachioradialis* ^a^	Böhmer et al. (2020)
*m. tensor fasciae antebrachii*	*m. epitrochlearis*	Böhmer et al. (2020)
	*Dorsi‐epitrochlearis*	Mackintosh (1875)
*m. triceps brachii caput longum*	*Triceps longus*	Mackintosh (1875)
*m. triceps brachii caput laterale*	*Triceps externus*	Mackintosh (1875)
*m. triceps brachii caput mediale*	*Triceps internus*	Mackintosh (1875)
*m. triceps brachii caput mediale* *m. triceps brachii caput accessorium*	*Triceps brachii caput mediale*	Böhmer et al. (2020)
*m. anconeus*	*m. anconeus lateralis*	Present study
*m. anconeus medialis* (Present study). Muscle without name at the NAV.	*Anconeus internus*	Mackintosh (1875)
	*Triceps brachii caput accessorium*	Böhmer et al. (2020)
	*Triceps brachii caput mediale accessorium* (*m. epitrochleo‐anconeus*, *m. anconeus internus*)	Tarquini et al. (2023)
*m. brachioradialis*	*Supinator radii longus*	Mackintosh (1875)
*m. extensor carpi radialis*	*Extensor carpi radialis longior* and *Extensor carpi radialis brevior*	Mackintosh (1875)
	*Extensor carpi radialis longus* and *Extensor carpi radialis brevis*	McClearn (1985), Böhmer et al. (2020) and Tarquini et al. (2023)
*m. supinator*	*Supinator radii brevis*	Mackintosh (1875)
*m. extensor digitorum communis*	*Extensor digitorum longus*	Mackintosh (1875)
*m. extensor digitorum lateralis*	*Extensor minimi digiti*	Mackintosh (1875)
*m. extensor carpi ulnaris*	*Extensor carpi radialis* ^a^	Mackintosh (1875)
*m. abductor digiti I longus*	*Extensor ossis metacarpi pollicis*	Mackintosh (1875)
	*Abductor pollicis longus*	McClearn (1985)
	*Extensor pollicis*	Böhmer et al. (2020)
*m. extensor digiti i* *m. extensor digiti II*	*Extensor secundi internodii pollicis et indicis*	Mackintosh (1875)
	*Extensors pollicis longus* and *indicis propius*	McClearn (1985)
	*m. extensor digiti I and II*	Böhmer et al. (2020)
	*m. extensor digiti I et II*	Tarquini et al. (2023) and present study

^a^ Editing errors of Mackintosh (1875) and Böhmer et al. (2020).

Based on the evolutionary history of AM in chordates, it could have derived in Reptilia from the *m. flexor antebrachii et carpi ulnaris* of amphibians (Diogo et al. 2018) (Table 6). However, in a posterior embryological study, the AM was a unique mammalian muscle since a homologous muscle was not found in the embryological development of reptiles (Smith‐Paredes et al. 2022). The AM derives from the proximal end of the ulnar lobe of the flexor subdivision in mammals, from which the FCU also forms (Smith‐Paredes et al. 2022). As a result, based on the topology, innervation, and the embryological evidence in mammals, the AM is not a head of TB in *N. nasua* (Table 6).

**TABLE 6:** Derivation hypotheses of the dorsal and ventral divisions from the last common ancestor of Amniota until *Nasua nasua* (muscles of the brachium and craniolateral antebrachium). The first two columns (Dorsal musculature and LCA of Amniota) are based on the embryological study of Smith‐Paredes et al. (2022). The next columns are based on that reported in mammals by several authors (Diogo et al. 2018; Gambaryan et al. 2015; Vélez García et al. 2024; Vélez‐García, Carrión Blanco, et al. 2023; Vélez‐García et al. 2021). Black arrows indicate the derivation hypotheses based on Diogo et al. (2018). Blue arrows indicate the derivation hypotheses based on Smith‐Paredes et al. (2022). Red arrows indicate the derivation hypotheses based on the present study. AbDIL, *m. abductor digiti I longus*; AL, *m. anconeus lateralis*; AM, *m. anconeus medialis*; B, *m. brachialis*; BB, *m. biceps brachii*; BR, *m. brachioradialis*; CB, *m. coracobrachialis*; CBb, *m. coracobrachialis brevis*; CBl, *m. coracobrachialis longus*; ECR, *m. extensor carpi radialis*; ECRB, *m. extensro carpi radialis brevis*; ECRL, *m. extensor carpi radialis longus*; ECU, *m. extensor carpi ulnaris*; EDC, *m. extensor digitorum communis*; EDI, *m. extensor digiti I*; EDII, *m. extensor digiti II*; EDI‐II, *m. extensor digiti I et II*; EDL, *m. extensor digitorum lateralis*; FCU, *m. flexor carpi ulnaris*; TBa, *m. triceps brachii caput accessorium*; TBLa, *m. triceps brachii caput laterale*; TBLo, *m. triceps brachii caput longum*; TBm, *m. triceps brachii caput mediale*; TFA, *m. tensor fasciae antebrachii*; S, *m. supinator*.

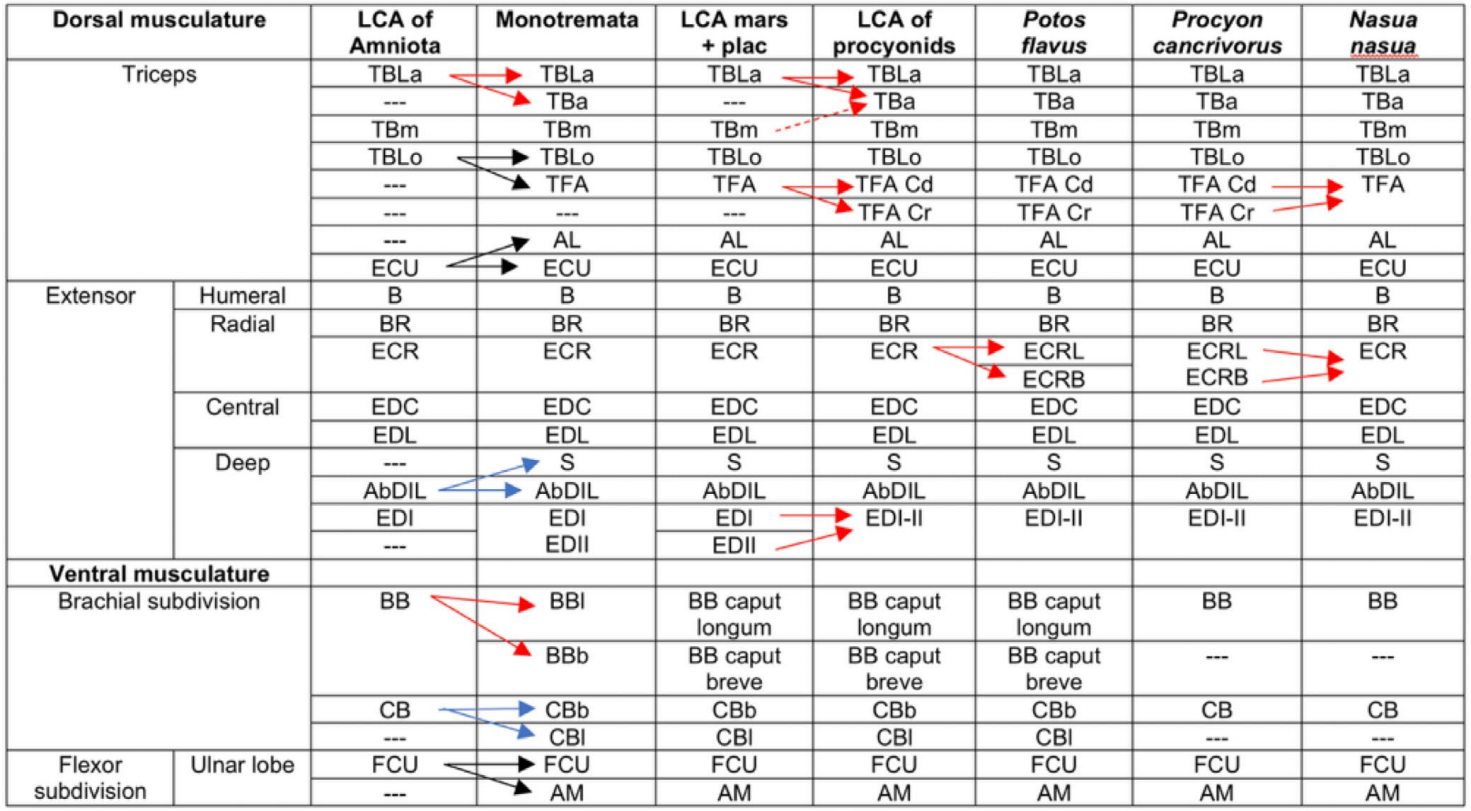

*Note:* The first two columns (Dorsal musculature and LCA of Amniota) are based on the embryological study of Smith‐Paredes et al. (2022). The next columns are based on that reported in mammals by several authors (Diogo et al. 2018; Gambaryan et al. 2015; Vélez García et al. 2024; Vélez‐García, Carrión Blanco, et al. 2023; Vélez‐García et al. 2021). Black arrows indicate the derivation hypotheses based on Diogo et al. (2018). Blue arrows indicate the derivation hypotheses based on Smith‐Paredes et al. (2022). Red arrows indicate the derivation hypotheses based on the present study.

Abbreviations: AbDIL, *m. abductor digiti I longus*; AL, *m. anconeus lateralis*; AM, *m. anconeus medialis*; B, *m. brachialis*; BB, *m. biceps brachii*; BR, *m. brachioradialis*; CB, *m. coracobrachialis*; CBb, *m. coracobrachialis brevis*; CBl, *m. coracobrachialis longus*; ECR, *m. extensor carpi radialis*; ECRB, *m. extensro carpi radialis brevis*; ECRL, *m. extensor carpi radialis longus*; ECU, *m. extensor carpi ulnaris*; EDC, *m. extensor digitorum communis*; EDI, *m. extensor digiti I*; EDII, *m. extensor digiti II*; EDI‐II, *m. extensor digiti I et II*; EDL, *m. extensor digitorum lateralis*; FCU, *m. flexor carpi ulnaris*; TBa, *m. triceps brachii caput accessorium*; TBLa, *m. triceps brachii caput laterale*; TBLo, *m. triceps brachii caput longum*; TBm, *m. triceps brachii caput mediale*; TFA, *m. tensor fasciae antebrachii*; S, *m. supinator*.

### 4.5 Comparative Arterial Supply

The collateral radial artery (CRA) originated from the caudal circumflex humeral artery (CdCHA) and coursed between TBa and TBLa in *N. nasua* , similar to *Procyon cancrivorus* (Vélez García et al. 2024), *Potos flavus* (Vélez‐García, Carrión Blanco, et al. 2023), *A. melanoleuca* (Davis 1964), *Felis catus* (Barone 2021; Vélez García et al. 2026), and sometimes in *Canis lupus familiaris* (Barone 2021; Hermanson et al. 2020) and *Cerdocyon thous* (Vélez et al. 2018). In this latter species, it can also originate from the cranial circumflex humeral artery (CrCHA) and pass between TBa and TBm (Vélez et al. 2018). In *N. nasua* , we identified the typical origin of CRA in procyonids, which facilitated differentiation of TBa from the TBLa when these were fused in one case (see Figure S2), similar to one case reported in *P. cancrivorus* (Vélez García et al. 2024). The vessels are known to participate in muscle splitting during limb development (Tozer et al. 2007), suggesting that CRA may contribute to the separation of TBa and TBLa in procyonids. The formation of several bicipital arteries in *N. nasua* has also been found in *P. flavus* (Vélez‐García, Carrión Blanco, et al. 2023) and *P. cancrivorus* (Vélez García et al. 2024), while in other species, this arrangement normally does not occur (Barone 2021; Davis 1964; Hermanson et al. 2020; Vélez García et al. 2026; Vélez et al. 2018). The superficial brachial artery (SBA) when coursing medially and distally to the belly of BB, provided several branches to BB and superficial antebrachial muscles in *N. nasua* , consistent with other procyonids (Perdomo‐Cárdenas et al. 2021; Vélez García et al. 2024; Vélez‐García, Carrión Blanco, et al. 2023) and the ursid *A. melanoleuca* (Davis 1964). Distally, the radial artery (RA) supplied the BR in *N. nasua* , a feature that differs from other procyonids.

### 4.6 Functional and Ecological Interpretations

In this section, we complement the functional and ecological interpretations of previous studies (Böhmer et al. 2019; McClearn 1985; Tarquini et al. 2023). The distinctive unique insertion of BB onto the radial tuberosity and the presence of a bicipitoradial bursa could be anatomical adaptations that enhance its role as another supinator muscle in *N. nasua* . This arrangement facilitates elbow flexion and lateral rotation of the radius, while the bursa minimizes tendon friction on the radius during pronation. Additional vascular and lymphatic adaptations, including multiple bicipital arteries and a brachial lymph node, could indicate that the muscle has elevated metabolic and drainage demands. Functionally, the BB should act synergistically with S and BR to generate powerful supination. The proximal insertion and bipennate shape of BB and B increase the velocity and strength in elbow flexion, respectively. While the distal insertion of BR and ECR only increases the strength in elbow flexion. The combined actions should optimize elbow mobility, enabling *N. nasua* to perform precise supination and flexion during climbing and repetitive flexion during digging. The proximal fusion of the ECR bellies in *N. nasua* could represent an intraspecific functional advantage in elbow flexion of *N. nasua* specimens comparatively with those specimens that are described with an independent *m. extensor carpi radialis brevis* by several authors (Böhmer et al. 2020; McClearn 1985; Tarquini et al. 2023). This is because the *m. extensor carpi radialis brevis* should not be an elbow flexor due to its distal origin.

Elbow extension in *N. nasua* is generated by TFA, the four heads of TB, AL and AM. The presence of two tricipital bursae (cranial and caudal) within the tendons of TB may represent an anatomical adaptation that reduces friction against the olecranon tuber during repetitive extension. Functionally, the development of AM may indicate that its complementary function is the medial stabilization of the elbow due to the pronounced medial epicondyle of the humerus in this species (Tarquini et al. 2019). The evolutionary derivation of AM from the caudomedial antebrachial group also could indicate a specialization to counteract medial elbow stresses during contraction of caudomedial antebrachial muscles. Ecologically, these modifications are significant since climbing requires stable elbow extension to support the body trunk while advancing along tree branches, whereas digging demands repetitive extension to displace soil and recover prey.

Carpal extension is mainly performed by ECR and complementarily by ECU, while the digital extension is performed by EDC, EDL, EDI‐II and AbDIL (Hermanson et al. 2020). Although McClearn (1985) and Böhmer et al. (2020) classified the ECU as an accessory elbow flexor, its distal origin from the lateral epicondyle and proximal fusion with the caudolateral aspect of the elbow joint capsule indicate that it does not contribute to elbow flexion. Instead, the ECU should retract the elbow joint capsule laterally together with the AL, preventing impingement during extension. Its primary function lies in carpal extension or flexion depending on the joint position, while its synovial bursa protects the tendon against friction with the ulna during carpal flexion. The ECU should also contribute to lateral deviation of the manus, a movement that antagonizes the medial deviation produced by AbDIL (Hermanson 2020). Functionally, this bidirectional control of carpal deviation is essential during climbing, where precise positioning of the manus secures grip on irregular surfaces, and during digging, where controlled deviation stabilizes the thoracic limb against soil resistance. Thus, the integration of brachium and craniolateral antebrachium muscles reflects an evolutionary balance between mechanical efficiency and joint protection, enabling *N. nasua* to perform arboreal, cursorial, and digging behaviours with high versatility. Such adaptations allow *N. nasua* to exploit diverse habitats, ranging from arboreal environments rich in fruits to terrestrial substrates where invertebrates and other prey are uncovered.

### 4.7 Limitations of the Study and Further Considerations

Most carcasses used in this study were roadkill specimens, thus fractures in some thoracic limb bones were observed in several specimens. However, the first dissected specimen that died of natural causes (NnS1) allowed us to obtain an anatomical basis to identify the attachment areas of affected muscles in roadkill specimens. The main limitation was the identification of the arterial distribution of most specimens, since the latex did not pass through the thoracic limb arteries due to ruptures of the aorta (NnS4–NnS8). Although we observed intra‐ and interspecific differences that, in further studies with more specimens, can be better elucidated and associated with muscle development.

The comparison with species of Feliformia is part of an ongoing separate part of our project. The homologies and evolutionary remarks of the present study were based on direct gross dissections and the embryological study of Smith‐Paredes et al. (2022), and although these authors used two distant phylogenetic mammals (a marsupial and a rodent) to the order Carnivora, most homologies could be determined by comparative anatomy. Therefore, the present work provides a solid anatomical foundation for future embryological investigations in the order Carnivora that allow confirming the muscle homologies of the brachium and craniolateral antebrachium muscles.

## 5 Conclusions

The homologies of most muscles of brachium and craniolateral antebrachium could be established in *Nasua nasua* based on the topology, innervation, anatomical variants and the most recent embryological study in amniotes. Several highlights were observed from this study, which differed from the literature available in this species. Among them, the cranial part of TFA of non‐*Nasua* procyonids is not lost in *N. nasua* but fused to the caudal part of TFA. The TB has four heads, and TBa could be potentially more derived from TBLa than TBm. The AM is an independent muscle, and its evolutionary derivation from the antebrachial musculature mass in mammals confirms that it is not a head of TB. The BB has only one head and the typical radial insertion of most caniforms. The B preserves a typical ulnar insertion of arctoids. The ECR was undivided proximally and only separated into two bellies (*longus* and *brevis*) from the middle third of the antebrachium. The muscle maps were mainly reconstructed to the origins of B, AM, ECR and AbDIL. The arteries to supply the brachium and craniolateral antebrachium muscles had a typical pattern of procyonids.

## Author Contributions

J.F.V.G. and M.A.M. performed the conception and design of the study. J.F.V.G. and R.A.C.B. acquired the data. J.F.V.G. interpreted the data and wrote the manuscript. All authors approved the last version of the manuscript.

## Funding

Universidade de São Paulo, Universidade Federal de Catalão, Universidad del Tolima and CAPES (Coordenação de Aperfeiçoamento de Pessoal de Nível Superior, Bolsa no. 88887.685526/2022‐00).

## Conflicts of Interest

The authors declare no conflicts of interest.

## Supporting information


**Figure S1:** Lateral views of the left brachium of a *Nasua nasua* after remove the *m. brachialis* (B) from the cranial insertion to observe the free zone of origin in the humerus (a, b) and the bipennate form of B (c).
**Figure S2:** Lateral views of the right brachium of a *Nasua nasua* (NnS7). (a) The *caput laterale* of *m. triceps brachii* (TBLa) was retracted cranially to observe its shared fibres with the *caput accessorium* of *m. triceps brachii* (TBa), and the passage of collateral radial artery (CRA) between both TBLa and TBa; (b) The TBLa was retracted caudally to observe the branch of the radial nerve (RN) perforating the TBa to innervate TBLa; (c) The AM was removed from its origin and retracted caudally to observe its innervation by two branches of the ulnar nerve (UN); AL, *m. anconeus lateralis*; TBa + TBLa, shared fibres between TBa and TBLa; TBLaa, accessory head of TBLa; TBm, *caput mediale* of *m. triceps brachii*. Scale bars: 10 mm.
**Figure S3:** Lateral (a, b) and caudal (c) views of the right thoracic limb of a *Nasua nasua* at the level of the elbow to observe the cranial and caudal tricipital bursae. AM, *m. anconeus medialis*; AL, *m. anconeus lateralis*; B, *m. brachialis*; TB, *m. triceps brachii*; TBa, *caput accessorium* of TB; TBLa, *caput laterale* of TB; TBLo, *caput longum* of TB; TBm, *caput mediale* of TB. Scale bars: 10 mm.
**Figure S4:** Cranial view of the right brachium of a *Nasua nasua* after retracting laterally the *m. brachioradialis* (BR) to observe the shared fleshy fibres of *m. brachialis* (B) to BR, and between them is passing the deep branch of the radial nerve (RN^db^). RCA, radial collateral artery; TBa, *caput accessorium* of *m. triceps brachii*; TBLa, *caput laterale* of *m. triceps brachii*.
**Figure S5:** Cranial views of right thoracic limbs of *Nasua nasua* at the level of the elbow to observe the innervation and arterial supply to *m. extensor carpi radialis* (ECR) (a, b) and a longitudinal section of ECR to observe the proximal fusion of both bellies (c). EDC, *m. extensor digitorum communis*.
**Figure S6:** Dorsal view of a right manus of *Nasua nasua* where the belly of *m. extensor digitorum communis* (EDC) only formed three tendons and the most medial tendon formed the tendon to the digits II and III.
**Figure S7:** Lateral views of right antebrachii of *Nasua nasua* to observe the fusion of the tendon of origin of *m. extensor carpi ulnaris* (ECU) with the elbow joint capsule (a, b), and a lateral view of the distal part of a right antebrachium and manus where the insertion tendon of ECU was retracted caudally to observe its synovial bursa on the styloid process of ulna (c and d).
**Figure S8:** Lateral deep views of right antebrachii of *Nasua nasua*. (a) The *m. extensor digiti I et II* (EdI‐II) was retracted caudally to observe its fleshy origin from the tendinous origin of the caudal extreme of *m. abductor digiti I longus* (AbDIL). (b, c) The AbDIL were retracted caudally to observe its fleshy origin from the interosseus antebrachial ligament (IOAL) and membrane (IOAM) and its arterial supply by perforating branches of the caudal interosseus artery (CdIOA).

## Data Availability

The data that support the findings of this study are available in the Supporting Information of this study.
