# Supplementary material for: Evolutionary Inferences Based on Thoracic Limb Muscles of South American Coati (Nasua nasua): Brachium and Craniolateral Antebrachium
Source: Anat Histol Embryol. 2026 Oct 16;55(6):e70182. doi: 10.1111/ahe.70182 (PMC13673571; doi:10.1111/ahe.70182)
Supplement: Supplementary file 1 [file AHE-55-e70182-s001.pdf]

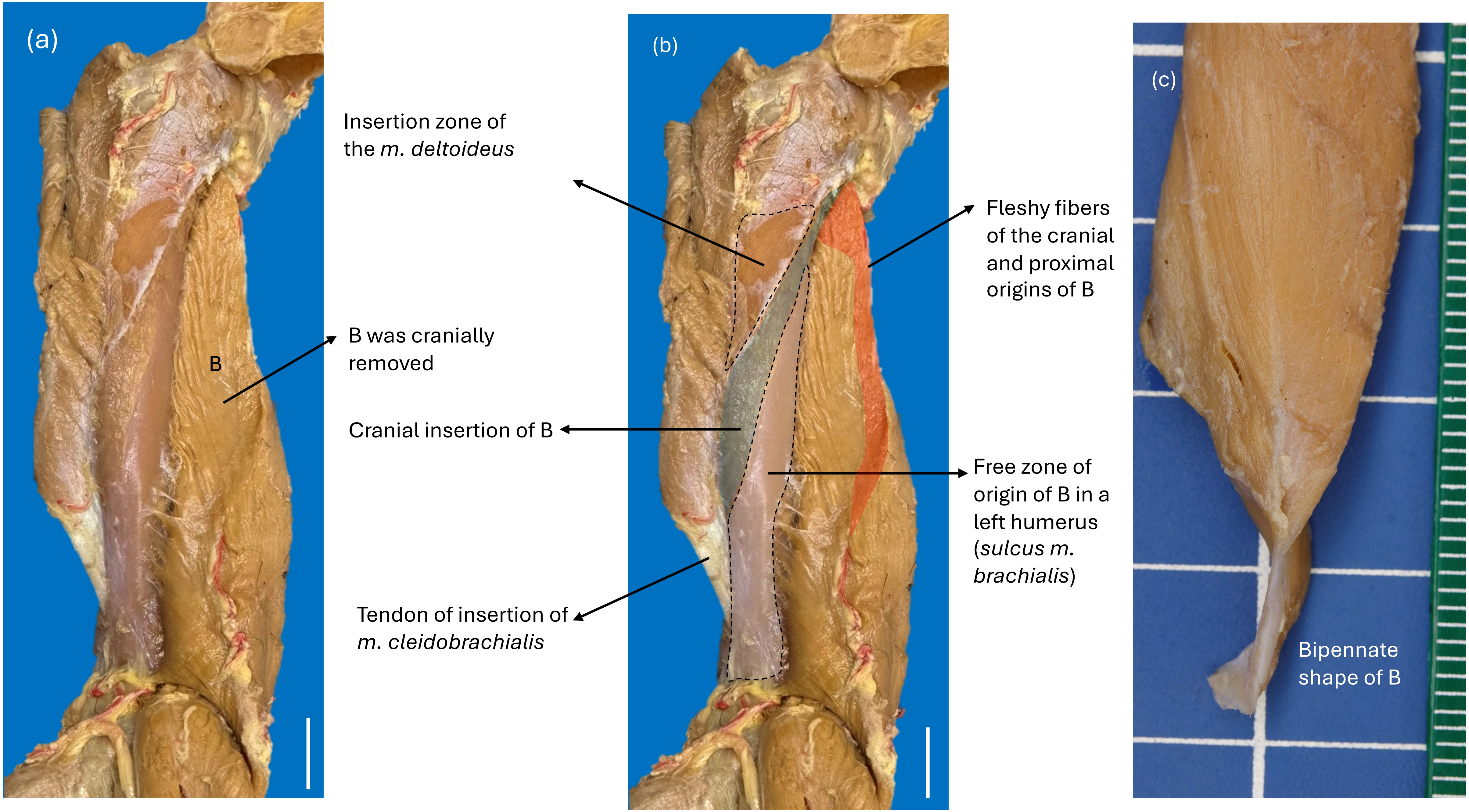
(a)
B
B was cranially removed
Insertion zone of the *m. deltoideus*
Cranial insertion of B
Tendon of insertion of *m. cleidobrachialis*
(b)
Fleshy fibers of the cranial and proximal origins of B
Free zone of origin of B in a left humerus (*sulcus m. brachialis*)
(c)
Bipennate shape of B

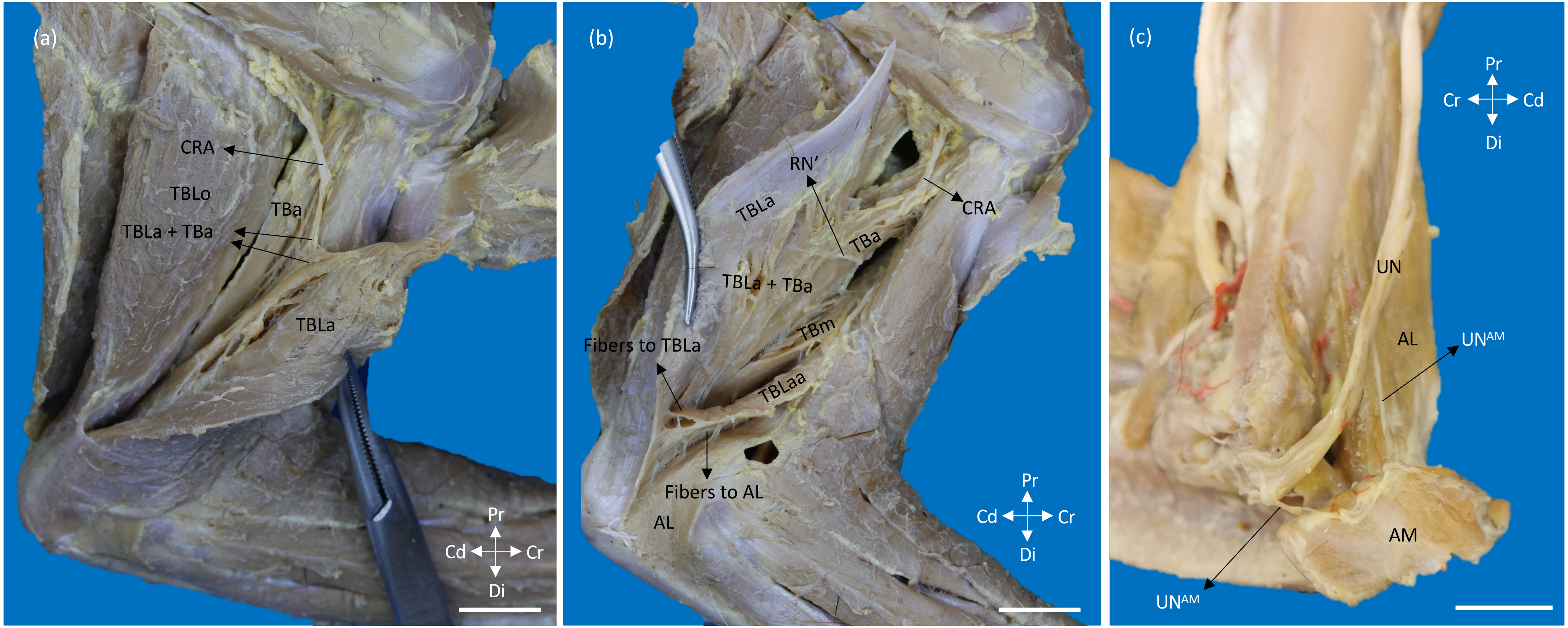
(a)
CRA
TBLo
TBa
TBLa + TBa
TBLa
Pr
Cd
Cr
Di
(b)
RN’
TBLa
CRA
TBa
TBLa + TBa
TBm
Fibers to TBLa
TBLaa
Fibers to AL
AL
Pr
Cd
Cr
Di
(c)
Pr
Cr
Cd
Di
UN
AL
UN^AM
AM
UN^AM

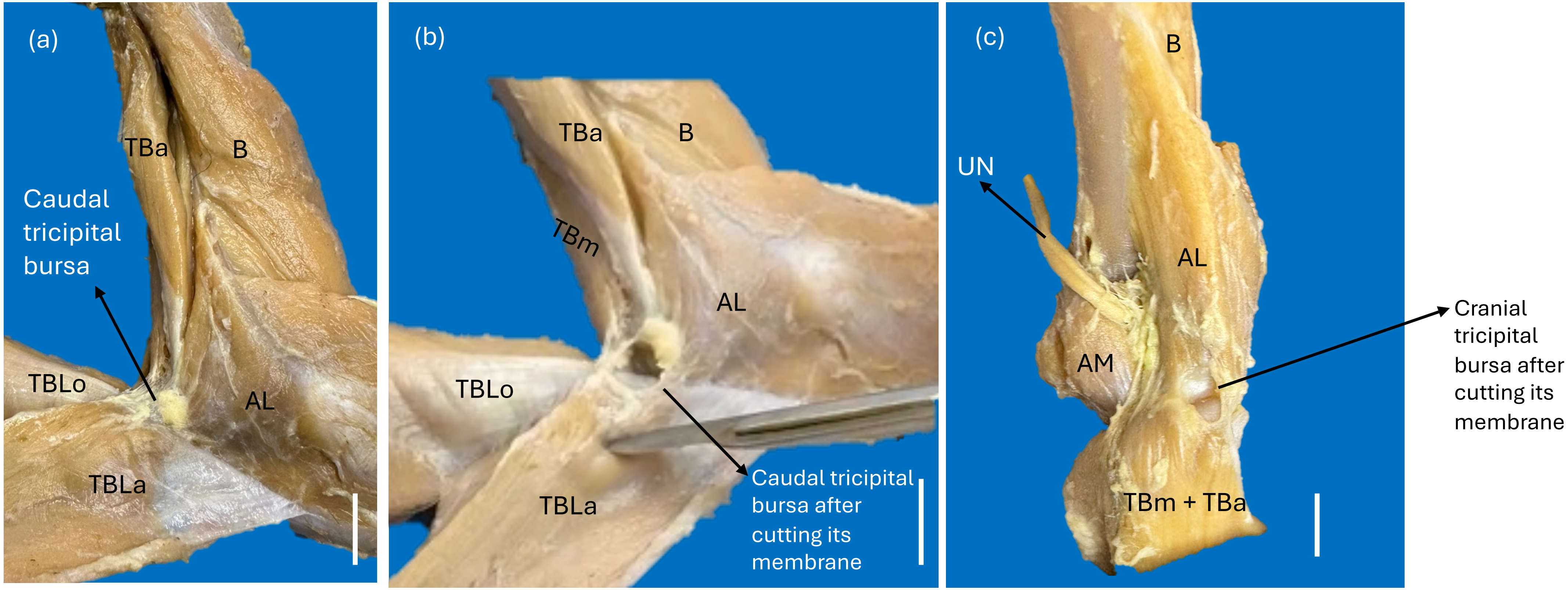

(a)
TBa
B
Caudal tricipital bursa
TBLo
AL
TBLa
(b)
TBa
B
TBm
AL
TBLo
TBLa
Caudal tricipital bursa after cutting its membrane
(c)
B
UN
AL
AM
TBm + TBa
Cranial tricipital bursa after cutting its membrane

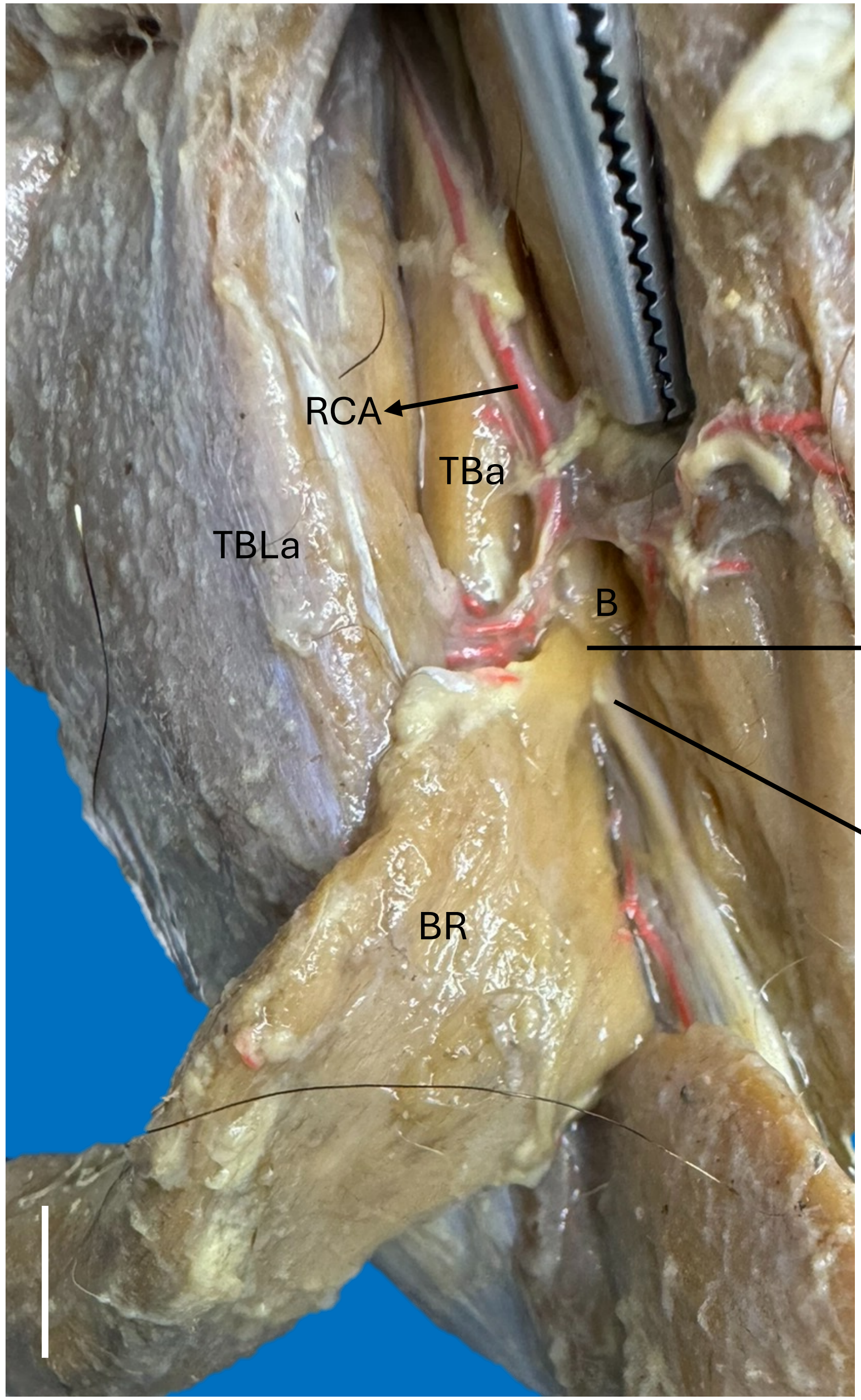

Shared fleshy fibers of B to BR

The deep branch of radial nerve (RN^{db}) is passing through the shared fleshy fibers between B and BR

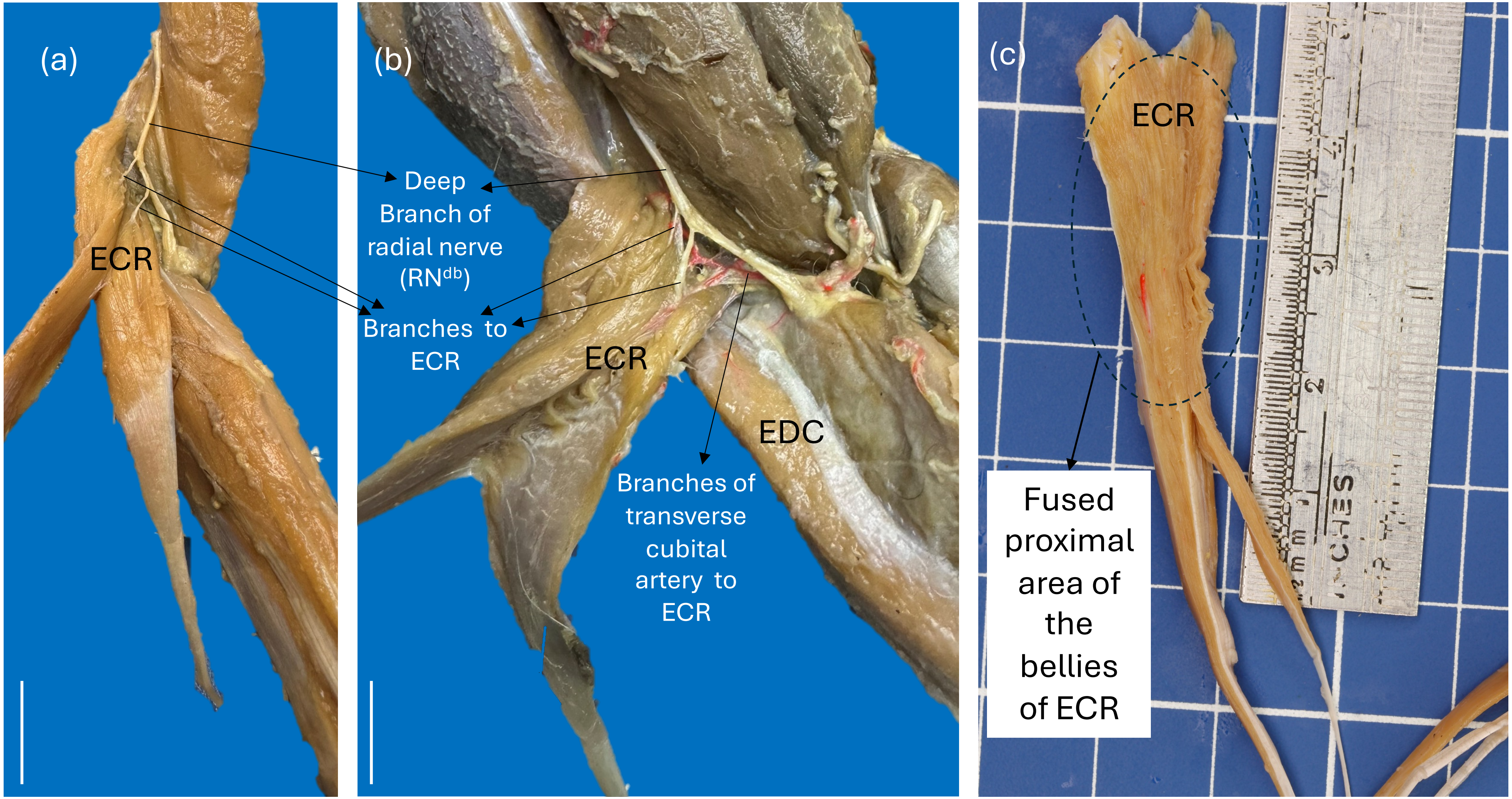

(a)
ECR
(b)
Deep Branch of radial nerve (RNdb)
Branches to ECR
ECR
EDC
Branches of transverse cubital artery to ECR
(c)
ECR
Fused proximal area of the bellies of ECR

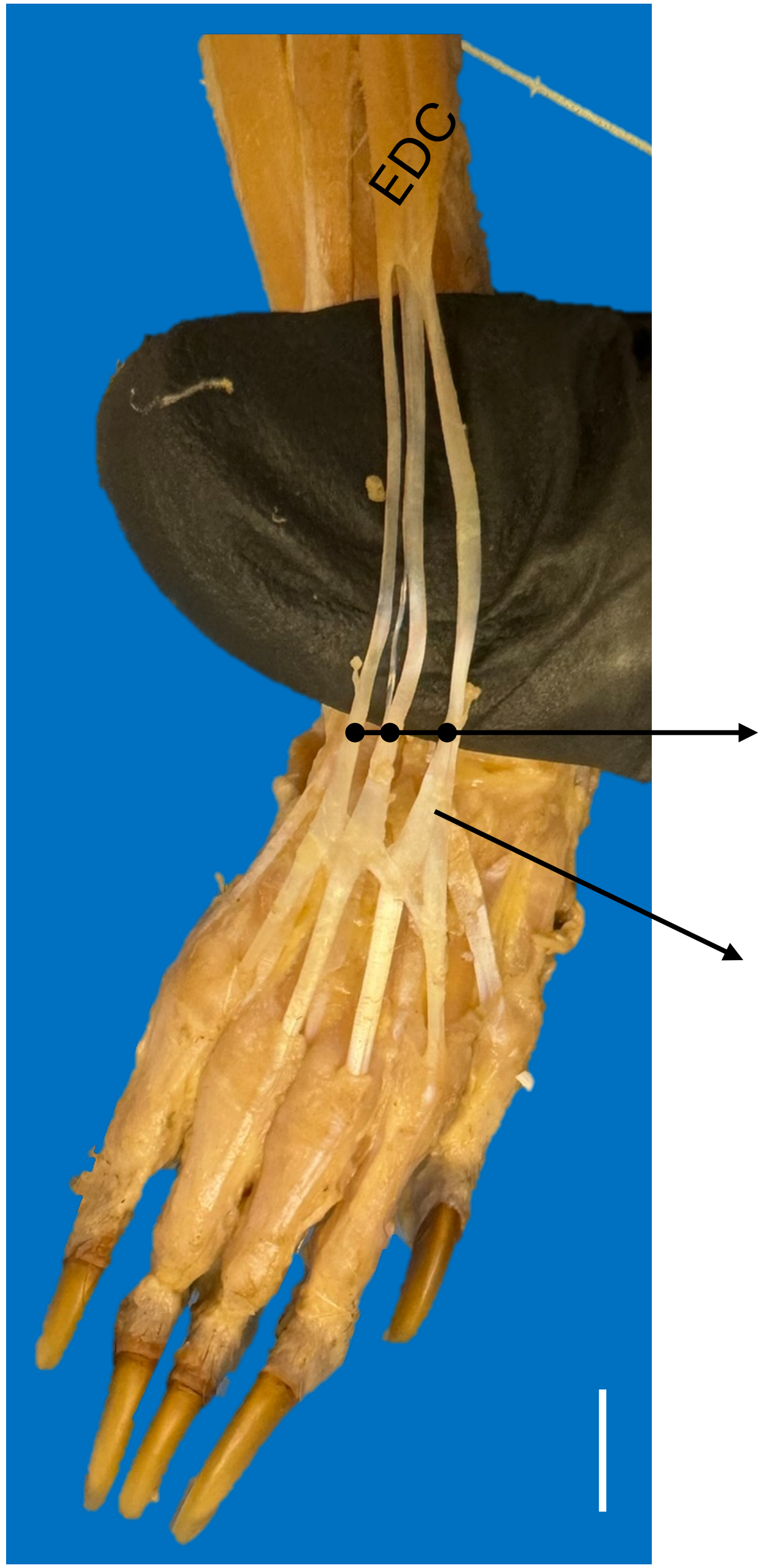

The belly of EDC only formed three tendons

The most medial tendon formed the tendons to the digits II and III

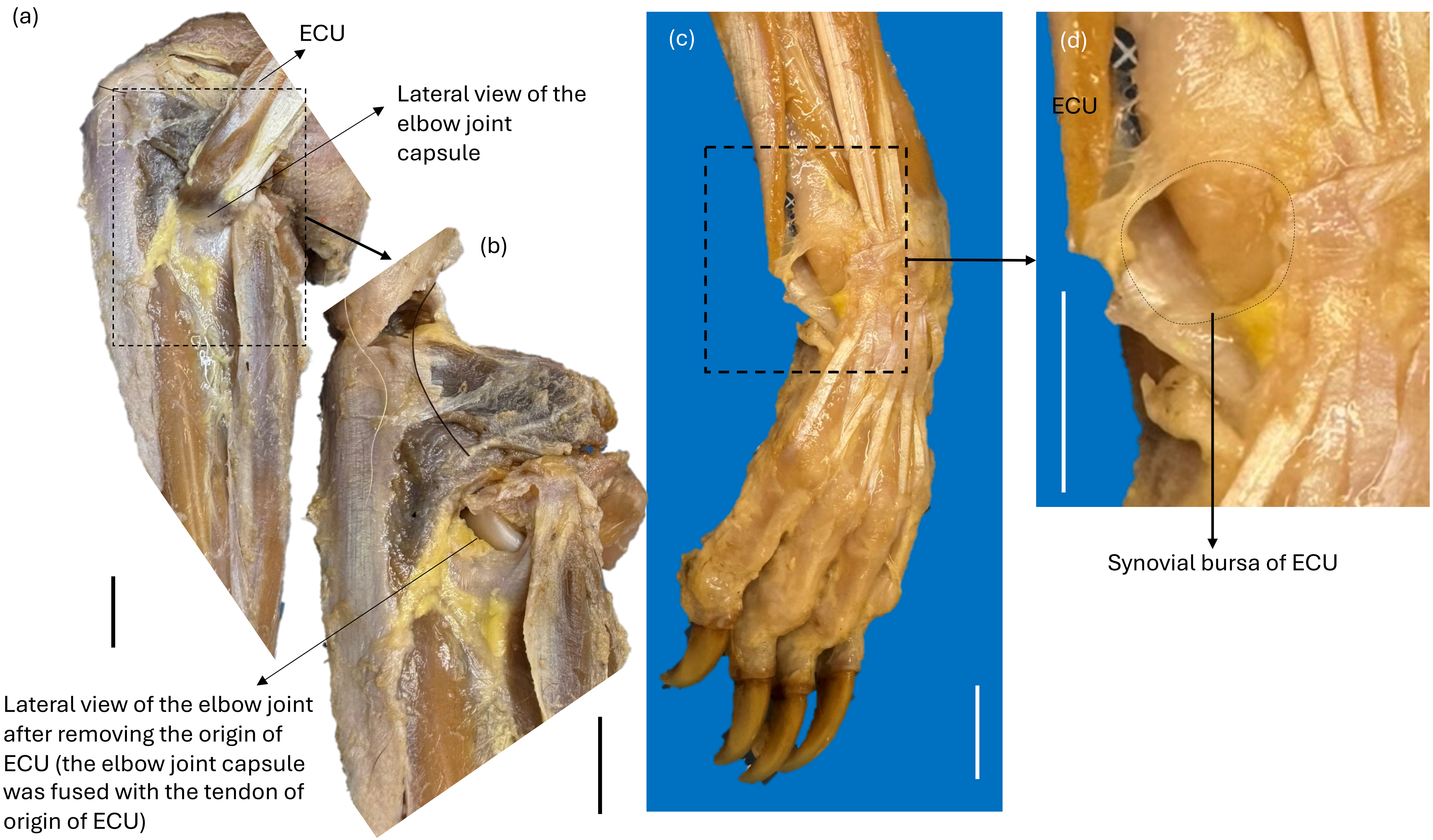
(a)
ECU
Lateral view of the elbow joint capsule
(b)
Lateral view of the elbow joint after removing the origin of ECU (the elbow joint capsule was fused with the tendon of origin of ECU)
(c)
(d)
ECU
Synovial bursa of ECU

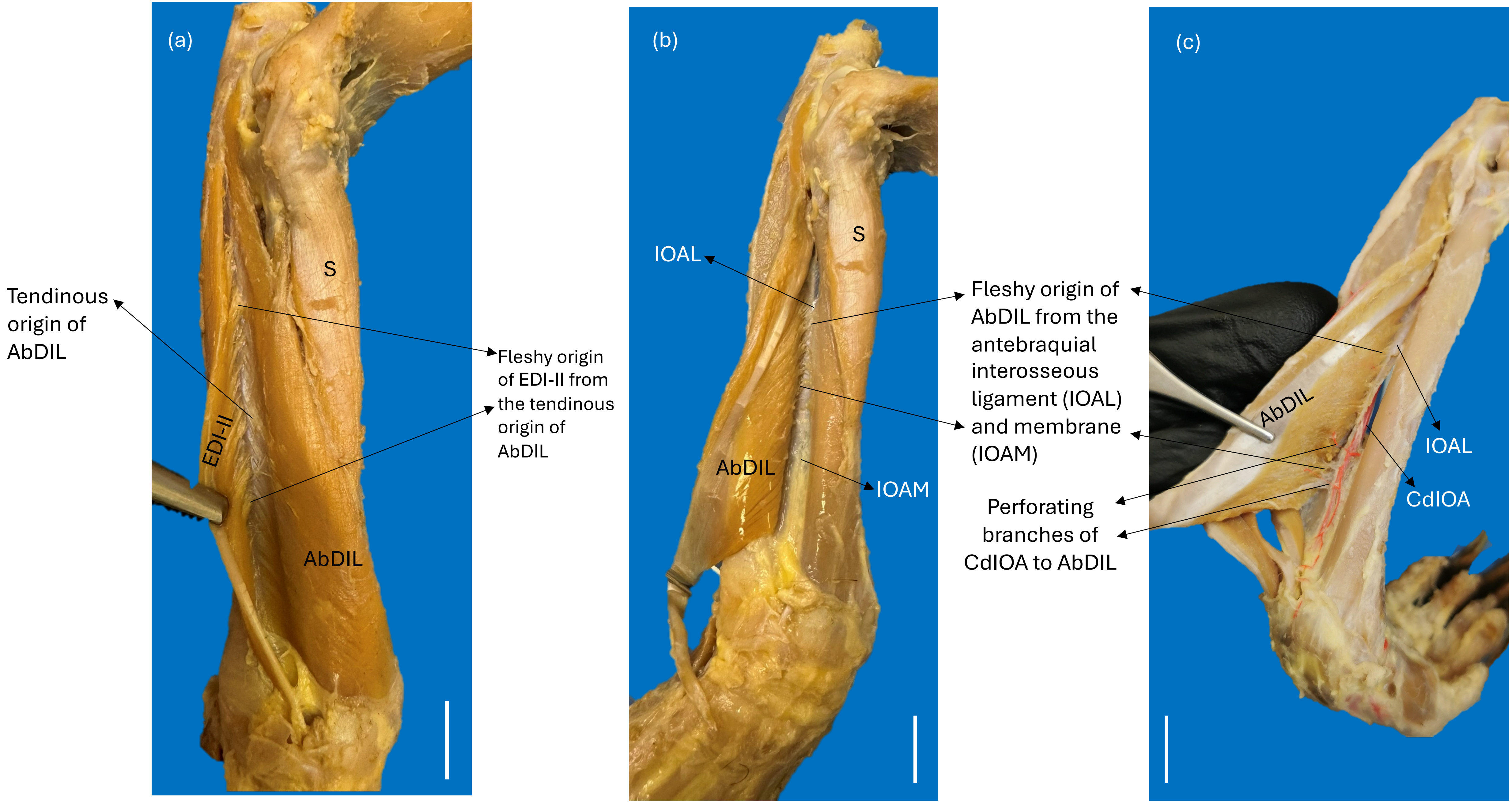
(a)
S
Tendinous origin of AbDIL
Fleshy origin of EDI-II from the tendinous origin of AbDIL
EDI-II
AbDIL
(b)
S
IOAL
AbDIL
IOAM
Fleshy origin of AbDIL from the antebraquial interosseous ligament (IOAL) and membrane (IOAM)
Perforating branches of CdIOA to AbDIL
(c)
AbDIL
IOAL
CdIOA
